# Removal of pharmaceuticals from nitrified urine by adsorption on granular activated carbon

**DOI:** 10.1016/j.wroa.2020.100057

**Published:** 2020-06-02

**Authors:** Isabell Köpping, Christa S. McArdell, Ewa Borowska, Marc A. Böhler, Kai M. Udert

**Affiliations:** a Eawag, Swiss Federal Institute of Aquatic Science and Technology, 8600 Dübendorf, Switzerland; b ETH Zürich, Institute of Environmental Engineering, 8093 Zürich, Switzerland

**Keywords:** Source separation, Organic micropollutants, Resource recovery, Recycling fertilizer, Carbon usage rate

## Abstract

Nitrification and distillation of urine allow for the recovery of all nutrients in a highly concentrated fertilizer solution. However, pharmaceuticals excreted with urine are only partially removed during these two process steps. For a sustainable and safe application, more extensive removal of pharmaceuticals is necessary. To enhance the pharmaceutical removal, which is already occurring during urine storage, nitrification and distillation, an adsorption column with granular activated carbon (GAC) can be included in the treatment train. We executed a pilot-scale study to investigate the adsorption of eleven indicator pharmaceuticals on GAC. During 74 days, we treated roughly 1000 L of pre-filtered and nitrified urine spiked with pharmaceuticals in two flow-through GAC columns filled with different grain sizes. We compared the performance of these columns by calculating the number of treated bed volumes until breakthrough and carbon usage rates. The eleven spiked pharmaceuticals were candesartan, carbamazepine, clarithromycin, diclofenac, emtricitabine, hydrochlorothiazide, irbesartan, metoprolol, N_4_-acetylsulfamethoxazole, sulfamethoxazole and trimethoprim. At the shortest empty bed contact time (EBCT) of 25 min, immediate breakthrough was observed in both columns shortly after the start of the experiments. Strong competition by natural organic material (NOM) could have caused the low pharmaceutical removal at the EBCT of 25 min. At EBCTs of 70, 92 and 115 min, more than 660 bed volumes could be treated until breakthrough in the column with fine GAC. The earliest breakthrough was observed for candesartan and clarithromycin. On coarse GAC, only half the number of bed volumes could be treated until breakthrough compared to fine GAC. The probable reason for the later breakthrough with fine GAC is the smaller intraparticle diffusive path length. DOC and UV absorbance measurements at 265 nm indicated that both parameters can be used as indicators for the breakthrough of pharmaceuticals. In contrast to pharmaceuticals and DOC, the nutrient compounds ammonium, nitrate, phosphate, potassium and sulfate were not removed significantly. A comparison with literature values suggests that the amount of GAC needed to remove pharmaceuticals from human excreta could be reduced by nearly two orders of magnitude, if urine were treated on site instead of being discharged and treated in a centralized wastewater treatment plant.

## Highlights

• Onsite treatment of nitrified urine can remove pharmaceuticals more efficiently than centralized wastewater treatment.
• Activated carbon adsorbs pharmaceuticals but no significant amounts of nutrients from nitrified urine.
• Nearly complete pharmaceutical removal was achieved for more than 660 bed volumes.

## Graphical abstract

**Figure:**
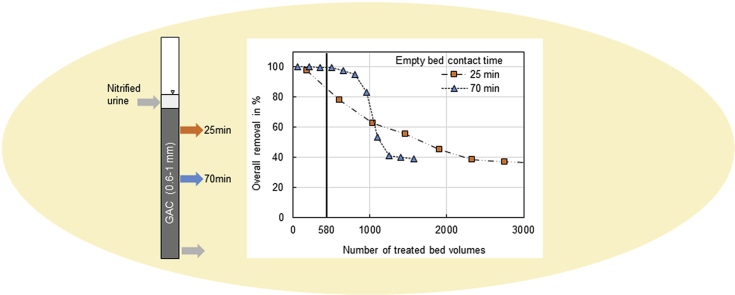

## 1 Introduction

Most nutrients from human metabolism are excreted with urine (Larsen and Gujer, 1996). Separate collection and treatment of urine has therefore been developed as an approach for preventing eutrophication, producing a valuable fertilizer and promoting sanitation in areas where no sewer-based sanitation is available (Udert et al., 2016). Maurer et al. (2006) proposed technologies for urine treatment and some of them have been tested in pilot scale (Udert et al., 2015). Since the review of Maurer et al. (2006), several new technologies have been explored, such as ammonia stripping in an electrochemical system (Kuntke et al., 2018) or the production of ammonium bicarbonate in a bio-electroconcentration system (Ledezma et al., 2017) The list of technologies for urine treatment is constantly growing. So far, nitrification and distillation of urine is probably the most thoroughly studied and tested technology for fertilizer production from urine (Fumasoli et al., 2016). It allows the recovery and stabilization of all nutrients contained in urine in a highly concentrated fertilizer solution. However, not only nutrients, but also other compounds could be recovered and concentrated, such as pharmaceuticals.

Separation of nutrients from pharmaceuticals is relevant to produce a safe fertilizer. Furthermore, on-site removal of pharmaceuticals from source-separated urine could be an effective way to reduce the discharge of organic micropollutants to the environment. Based on a literature review, Lienert et al. (2007a) estimated that 64% of the active ingredients are excreted with urine and the rest with feces.

Many processes for pharmaceutical removal from urine have been tested, all of them in laboratory experiments and most of them with synthetic solutions mimicking fresh or stored urine. Nevertheless, the results allow for a first comparison of the performance on pharmaceutical removal. Biological processes showed very different degradation efficiencies for various compounds. Oezel Duygan et al. (in prep.) examined the fate of twelve pharmaceuticals during aerobic biological treatment and anaerobic storage. In the nitrification step during which heterotrophic bacteria also degrade most of the bulk organic compounds (Udert and Wächter, 2012) atazanavir, ritonavir, darunavir and clarithromycin were rapidly degraded, while others, such as emtricitabine, trimethoprim, sulfamethoxazole, N_4_-acetylsulfamethoxazole, diclofenac, hydrochlorothiazide, atenolol and atenolol acid were only slowly degraded or not affected at all (Oezel Duygan et al., in prep.). After 50 days of storage under anaerobic conditions, more than 90% of hydrochlorothiazide was removed, while the overall removal for the other compounds was negligible. In another study, de Wilt et al. (2016) observed the removal of pharmaceuticals by biodegradation and photolysis with algae growth, while nutrients were recovered within the biomass. The authors reported high removal (60–100%) of diclofenac, ibuprofen, paracetamol and metoprolol and partial removal (30 and 60%) of trimethoprim and carbamazepine. Advanced oxidation processes with ozone (Dodd et al., 2008) or combinations of UV and H_2_O_2_ (Zhang et al., 2015) could only partially remove pharmaceuticals due to the reaction of the oxidants with other compounds of the urine matrix. Another set of studies tested the effect of membranes on pharmaceutical removal. Pronk et al. (2006) found that nanofiltration (NF) membranes rejected over 90% of pharmaceuticals from fresh urine. However, the NF membranes also rejected over 90% of the phosphate thus requiring a subsequent step to separate phosphate from pharmaceuticals. By including microfiltration or electrodialysis as a pretreatment step to nanofiltration, high pharmaceutical removal and longer operation times were achieved (Pronk et al., 2007). An intensively studied process on phosphorus recovery from urine is struvite precipitation (Ronteltap et al., 2007). Escher et al. (2006) reported that more than 99% of carbamazepine, diclofenac, ibuprofen and propranolol remained in the liquid phase so that the phosphorus product struvite contained negligible amounts of these pharmaceuticals. In addition, the biotests on estrogenicity and baseline toxicity showed that both effects were reduced by 98% in struvite. Furthermore, the two hormones 17α-estradiol (E2) and 17β-ethinylestradiol (EE2) could not be detected in struvite. Pharmaceuticals and hormonally active substances are concentrated in the effluent solution of the precipitation process. Depending on the local regulations, further processing of the effluent would be necessary to eliminate the organic micropollutants. Other researchers tested the adsorption of pharmaceuticals on anion exchange polymer resins (Landry and Boyer, 2013), on biochars (Solanki and Boyer, 2017) and on powdered activated carbon (PAC) (Oezel Duygan et al., in prep.). Results of the experiments with anion exchange polymer resins and biochars showed unwanted side-effects such as a co-occurring 20% removal of phosphate and nitrogen species (Solanki and Boyer, 2017) or a concomitant desorption of chloride (Landry and Boyer, 2013). The results of the study by Oezel Duygan and co-workers were promising. PAC dosage of 200 mg/L to biologically treated urine removed 90% of all tested compounds and the results motivated us to investigate pharmaceutical removal on activated carbon in more detail. In general, activated carbon is a popular adsorbent for pharmaceuticals for several reasons. First, adsorption has a low energy demand compared for example to oxidation processes. Second, activated carbon can be used in batch or continuous-flow reactors. Third, a wide range of reactor configurations are possible including mixed slurry or fixed bed, and, fourth, GAC can be used in convenient filter beds with the possibility to be reactivated and reused (Crittenden et al., 1999).

Any pharmaceutical removal process, including adsorption on activated carbon, requires regular maintenance such as replacement of the activated carbon to ensure a sufficient removal efficiency. Regular monitoring can help to optimize the replacement intervals. The most accurate measurements include the chemical analysis of micropollutants, but this approach is costly and time intensive. Measuring UV absorbance is a less expensive and simpler method. The use of the UV absorbance at 254 nm (UV_254_) was proposed by Altmann et al. (2016a) and Mailler et al. (2016) as a surrogate parameter to predict the overall removal of pharmaceuticals from wastewater. The dissolved organic carbon concentration (DOC) was also used as indicator for pharmaceutical removal from wastewater by adsorption on PAC (Altmann et al., 2014), on GAC (Meinel et al., 2015) and on micro-grained GAC (Mailler et al., 2016).

To our knowledge, removal of pharmaceuticals from real nitrified urine by adsorption on GAC has not been studied before. Based on results with GAC in municipal wastewater (e.g. Meinel et al., 2015) and PAC in nitrified urine (Oezel Duygan et al. in prep.), we set up the research hypothesis that adsorption on GAC in a flow-through filter allows nearly complete removal of pharmaceuticals from nitrified urine without losing significant amounts of nutrients. To test the hypothesis we

i. determined the individual and the overall removal of eleven pharmaceuticals depending on the run time of the GAC filter,
ii. investigated the influence of the EBCT on pharmaceutical removal,
iii. investigated the influence of the carbon grain size on pharmaceutical removal,
iv. calculated the treatment efficiency as carbon usage rates (CUR) and compared our results with the efficiency of advanced treatment of municipal wastewater,
v. tested whether nutrients were removed by the adsorption process and
vi. evaluated whether the DOC concentration or UV absorbance are reliable parameters to predict pharmaceutical removal with GAC.

## 2 Materials and methods

### 2.1 Experimental setup

Two identical columns were filled with two types of GAC with different grain sizes made from coconut shell (GCN 830, Norit Nederland BV, 3800, AC Amersfoort, The Netherlands). The fine and the coarse GAC had grain diameters between 0.6 and 1.0 mm and between 1.4 and 2.4 mm, respectively. Both fractions were retrieved manually by sieving the original material (median particle size 1.68 mm) with standard sieves. For more information on the GAC properties see Table S 1 in the supporting information (SI). The columns were made of PVC and had the following dimensions: total height 1900 mm, outer diameter 63 mm, inner diameter 53.6 mm and filtration area 22.6 cm^2^. GAC was filled into the columns to a total height of 64.5 and 64.3 cm with 1352 and 1328 g of the wetted coarse and fine GAC, respectively. Ball valves made of chromed brass for sampling were located at 5.5, 15.5, 20.5 and 25.5 cm in the coarse GAC column and at 5.3, 15.3, 20.3 and 25.3 cm in the fine GAC column. The empty bed contact times (EBCTs), which are calculated by dividing the bed volume of a column section by the volumetric flow, were on average 25, 70, 92 and 115 min (Table S 2, SI). Samples were taken from the overall cross section with a perforated stainless steel pipe inserted at each sampling height (Figure S 1, SI). The pipe and the valve were connected with a reduction nipple made of stainless steel. Twice a week samples were taken from the influent tank and all sampling points of the columns. The columns were wrapped in aluminum foil to prevent activity of phototrophic microorganisms. A scheme and picture of the experimental setup can be found Fig. S 2(SI).

### 2.2 Operation of the GAC columns

Before starting the experiment, the GAC columns were backwashed with tap water to remove fine carbon dust. With two peristaltic pumps (Ismatec™ Reglo Digital, Ismatec, Wertheim, Germany) nitrified urine was added to the upper part of the columns, where it infiltrated gravimetrically into the columns. The flow rate was controlled by weighing the effluent tanks every 48 h. If the flow rate differed by more than 10%, the pump rate was manually readjusted. To minimize the amount of particles pumped onto the columns, the nitrified urine in the influent tank was collected with a floating gauge. The two GAC columns ran continuously for 74 days and the inflow rates were sufficiently high to ensure that the GAC beds were completely submersed during operation. About 510 L of nitrified urine were treated in each column. During the operation of the GAC filters, the hydrostatic pressure rose continuously in both columns. We assume that this increase was caused by the accumulation of organic material, such as biofilm, and inorganic material, such as fine GAC particles, inside and on top of the GAC bed. Nevertheless, the maximum tolerable head loss was not reached during the 3 months of operation, so that backwashing was not necessary. At the end of the experiment, the supernatant on top of the filter bed had increased to 1460 and 1560 mL for coarse and fine GAC, respectively (Fig. S 4, SI). The corresponding head losses were 64.6 and 69.0 cm, respectively. The pumping rates were adjusted to maintain a constant flow rate of 5.1 ± 0.4 and 5.1 ± 0.5 mL/min for coarse and fine GAC, respectively (Fig. S 4, SI). The corresponding filter velocity was 0.14 ± 0.01 m/h for both columns. With a constant flow rate, the EBCTs were also nearly constant over time (Fig. S 5, SI). The Reynolds numbers (Re) for the columns with fine and coarse GAC were 0.08 and 0.17, respectively. Both values are close to 0.1, which is the recommended minimum Re for small scale GAC columns (MWH, 2012). More details are given in section 3.2 of the SI. The influent pH decreased from 6.9 at the beginning of the experiment to 5.9 at the end. After 30 days of operation, we decided to measure the pH at all sampling ports during sampling (Fig.S 6, SI). After the treatment with GAC, the urine had almost no color or odor (Fig. S 8, SI). All operational parameters and concentrations in the influent and the effluents are compiled in Table S 9 and S 10.

### 2.3 Urine and pharmaceuticals

We used partially nitrified urine from the pilot plant in Eawag’s main building as influent for the experiments (Fumasoli et al., 2016). To ensure constant influent concentrations during the experiment, we collected the total volume of about 1200 L urine beforehand, filtered it with a filter bag (pore size 50 μm) and measured the background pharmaceuticals concentrations (c_u,nitr_). As the background concentrations of Eawag’s urine were very low (see Table 1), we decided to spike a known amount of pharmaceuticals to reach levels to be expected for biologically treated urine (later on called reference urine). To calculate these levels, we made two assumptions. First, nitrification of urine results in the same relative degradation of pharmaceuticals as in biological treatment of municipal wastewater as seen by Oezel Duygan et al. (in prep.). Second, urine is 100 times more concentrated than wastewater, which is a conservative assumption since typical dilutions are often 200 times or more. On average 350 L of wastewater (Gujer, 2007) and 1.25 L of urine (Udert et al., 2006) are produced per person and day. To calculate the pharmaceutical concentrations in the reference urine (c_u,ref_), typical concentrations for biologically treated Swiss wastewater (c_ww__,bio_) (Götz et al., 2014) were multiplied with the relative excretion (${\text{e}}_{\text{urine}}$) from the human body via urine and multiplied with a dilution factor of 100 (see section 2.1, SI for the calculation).

**Table 1:** Concentrations of pharmaceuticals measured in biologically treated municipal wastewater (c_ww,bio_), in the envisaged reference urine (c_u,ref_), in nitrified and filtered urine before (c_u,nitr_) and after spiking (c_u,spike_). Details about the calculation can be found in section 2.1 (SI).

Compound		Type	Log D_OW_ (at pH 6)	Relative excretion via urine in %	Concentration in μg/L			
					c_ww,bio_	c_u,ref_	c_u,nitr_	c_u,spike_
Candesartan	CAN	Antihypertensive	0.91^b^	32^f^	0.40	12.7	0.50	11.0
Carbamazepine	CAR	Antiepileptic	2.77^a^	8^g^	0.58	4.49	0.30	5.42
Clarithromycin	CLA	Antibiotic	0.89^a^	76^f^	0.11	8.26	34.4	51.9^c^
Diclofenac	DCF	Analgesic	2.26^a^	100^f^	0.97	97.2	4.50	80.6
Emtricitabine	EMT	Virostatic	−0.73^b^	84^f^	0.28	23.6	2.55	2.57^d^
Hydrochlorothiazide	HCT	Diuretic	−0.58^a^	82^h^	1.25	102	3.65	84.5
Irbesartan	IRB	Antihypertensive	1.99^b^	9^h^	0.67	6.05	0.00	4.70
Metoprolol	MET	Beta blocker	−1.34^a^	64^g^	0.35	22.6	10.1	27.0
N_4_-Acetylsulfamethoxazole	NSMX	Metabolite	0.55^a^	100^f^	0.08	7.52	0.15	5.51
Sulfamethoxazole	SMX	Antibiotic	0.6^a^	100^g^	0.25	23.5	2.50	5.85^e^
Trimethoprim	TMP	Antibiotic	0.27^a^	100^f^	0.05	4.46	0.10	4.59

a Values from Kovalova et al. (2013).

b Predicted by ACD/Labs (www.chemspider.com) for pH 5.5.

c Although the concentration in the urine collected at Eawag was already high, CLA was spiked due to an error.

d EMT was not spiked because it was not available as standard when spiking was done.

e Less was spiked than envisaged due to an error during weighing in.

f Excretion rates calculated with Swiss Compendium of Medicines by Documed: www.compendium.ch, last accessed July 4, 2019.

g Calculated with excretion rates from Lienert et al. (2007b).

h Calculated with excretion rates from Lienert et al. (2007a).

We prepared a concentrated pharmaceutical mix, taking the pharmaceutical concentrations already present in the collected urine into account (section 2.2, SI). The mix was added to the urine to reach the expected concentrations of the reference urine (c_u,ref_). Later, pharmaceutical concentrations were measured to determine the actual concentrations (c_u,spike_). The relative excretion rates and concentrations used to calculate the pharmaceutical concentrations in the reference urine (c_u,ref_) are given in Table 1*.* For this study we decided to examine the removal of the following compounds: candesartan (CAN), carbamazepine (CAR), clarithromycin (CLA), diclofenac (DCF), emtricitabine (EMT), hydrochlorothiazide (HCT), irbesartan (IRB), metoprolol (MET), N_4_-acetylsulfamethoxazole (NSMX), sulfamethoxazole (SMX) and trimethoprim (TMP). These pharmaceuticals were chosen because they occur in high concentrations in Swiss (Singer et al., 2016) and European wastewater (Loos et al., 2013). Many of them are also indicator substances selected to evaluate the effectiveness of advanced wastewater treatment in Swiss wastewater treatment plants (WWTPs) (Bourgin et al., 2018). The selection includes compounds with a high tendency of adsorption to activated carbon, i.e. CAR, CLA, DCF, HCT and MET, and low tendency of adsorption to activated carbon, such as CAN and SMX (Kovalova et al., 2013).

### 2.4 Sampling and analysis of general parameters

Aliquots of 10 mL (of which 3 mL were separated for the analysis of pharmaceuticals) were taken at each sampling point twice a week during 74 days. When taking a sample, the first 5 mL were discarded, because the dead volume in the valve and the sampling tube was estimated to be 5 mL. To minimize the influence of the sampling procedure on the fluid dynamics inside the column, sampling ports were opened slowly and only partially. The samples were diluted with Nanopure® water (200 times for anions, 100 times for cations and 20 times for dissolved organic carbon) and filtered through glass microfiber filters (0.45 μm, MN GF-5, Macherey-Nagel, Düren, Germany). Samples for the analysis of the cations ammonium, potassium, sodium, calcium and magnesium were acidified with 1 mol/L nitric acid. Samples for the analysis of the cations and the anions nitrate, phosphate, sulfate and chloride, were measured with ion chromatography (881 compact IC pro, Metrohm, Herisau, Switzerland). Dissolved organic carbon (DOC) was measured with a TIC/TOC analyzer (IL550 OmniTOC, Hach-Lange, Berlin, Germany). The standard deviation for all chemical measurement methods was below 5%. Temperature, pH and conductivity in the influent and the effluents were measured in-situ with handheld instruments (WTW pH 340i, WTW GmbH, Weilheim, Germany). A list with all measured parameters can be found in the SI in Table S 3.

### 2.5 Chemical analysis of pharmaceuticals

Collected samples (aliquots of 3 mL) were kept frozen between sampling and analysis. Shortly before analysis, samples were thawed, diluted 100 times with Nanopure® water to minimize matrix effects, spiked with isotope-labeled internal standards and filtered. Pharmaceuticals were analyzed using an online solid phase extraction system combined with liquid chromatography coupled to a high resolution mass spectrometer (Q-Exactive^TH^ Plus, ThermoScientific, Massachusetts, United States), further called online-SPE-LC-HRMS, according to Bourgin et al. (2018). SPE cartridges used for enrichment contained Oasis® HLB sorbent (15 μm, Waters, USA), anion exchanger Strata X-AW, cation exchanger Strata X–CW (30 μm, Phenomenex, UK) and Env+ (Biotage, Sweden). For LC an Atlantis® T3 column (3.0 × 150 mm, particle size 3 μm, Waters, Massachusetts, United States) and for detection a HRMS using electrospray ionization (Q-Exactive® Plus, ThermoFisher Scientific, Massachusetts, United States) was used. The limit of quantification (LOQ), ranging between 0.04 and 1.00 μg/L and the relative recovery, commonly in the range of 80–120%, were determined in each series. More information about the chemical analysis and data processing can be found in the SI (see section S 4 and Tables S5 and S 6 and S 7). SMX was evaluated together with its main metabolite N_4_-acetyl-sulfamethoxazole (NSMX), because its back transformation during wastewater treatment was observed in several studies (Letzel et al., 2010, and Göbel et al., 2004).

### 2.6 Calculation of pharmaceutical removal and breakthrough

The removal of each compound at each sampling time *t* was calculated by comparing the effluent (${\text{c}}_{\text{eff},\text{t}}$) and the influent concentration (${\text{c}}_{\text{inf},\text{t}}$) (Equation (1)). The overall removal of all pharmaceuticals was calculated as arithmetic mean of the single compound removal efficiencies for each sampling time.

(1) $$\text{Removal}=\left(1-\;\frac{{\text{c}}_{\text{eff},\text{t}}}{{\text{c}}_{\text{inf},\text{t}}}\;\right)\cdot 100\;\left[\text{\%}\right]$$

Especially for long EBCTs and in the beginning of the experiment, when the adsorption capacity of the GAC was still high, the pharmaceutical effluent concentrations were below the LOQ of the analytical method. In most cases, the LOQ was below 2% of the influent concentration, except for EMT, where the LOQ was up to 20% of the influent concentration. If effluent concentrations were below LOQ the removal was defined as 100%. Breakthrough of pharmaceuticals was defined as the point when the removal dropped permanently below 98%.

### 2.7 Calculation of treated bed volumes and carbon usage rates

The number of treated bed volumes (n_BV_) can be used to compare the treatment efficiency independent of reactor sizes. To calculate n_BV_ the volume of the urine treated (V_treated_) is devided by the bed volume (V_b_) of the respective reactor section (Equation (2)). V_treated_ is calculated by multiplying the flow rate to the adsorber (Q) with the running time (t).

(2) $${\text{n}}_{\text{BV}}=\frac{{\text{V}}_{\text{treated}}}{{\text{V}}_{\text{b}}}=\frac{\text{Q}\cdot \text{t}}{{\text{V}}_{\text{b}}}\;\left[\frac{{\text{m}}^{3}}{{\text{m}}^{3}}\right]$$

To quantify the performance of the GAC columns, we calculated carbon usage rates (CUR) for a defined treatment goal. CUR is defined as the mass of GAC in the adsorber (m_GAC_) divided by the volume of treated urine *V*_*treated*_ (Equation (3)).

(3) $$\text{CUR}=\;\frac{{\text{m}}_{\text{GAC}}}{{\text{V}}_{\text{treated}}}\;\left[\frac{\text{mg GAC}}{\text{L}}\right]$$

CUR or n_BV_ are usually given for specific treatment goals. In this study, we compared the removal of pharmaceuticals with coarse and fine GAC for complete pharmaceutical removal. Breakthrough was defined as the time, when the pharmaceutical removal dropped permanently below 98%. When comparing our results with studies from municipal wastewater treatment, we used a treatment goal of 90% pharmaceutical removal, because municipal wastewater treatment does not aim for complete pharmaceutical removal.

### 2.8 Measurement of UV absorbance

UV absorbance measurements were done with a UV–Vis spectrophotometer (Agilent Cary 60, Agilent Technologies, Santa Clara, United States) in the range of 200–800 nm. Preliminary tests have shown that in nitrified urine it is difficult to use the wavelength of 254 nm, which is typically used in wastewater as surrogate for organic compounds. Nitrate, which is present in much higher concentrations in nitrified urine (in our case 2080 mg N/L) than in the effluent of WWTPs (about 10 mg N/L), strongly absorbs at wavelengths between 200 and 250 (section 5 and Figure S 3, SI). To prevent any influence of changes in the nitrate concentration on UV measurements (for details see Mašić et al., 2015), we chose a slightly higher wavelength of 265 nm. For UV–Vis analysis, all samples were diluted by a factor of 10.

## 3 Results and discussions

### 3.1 Influence of empty bed contact time

**Fig. 1:**
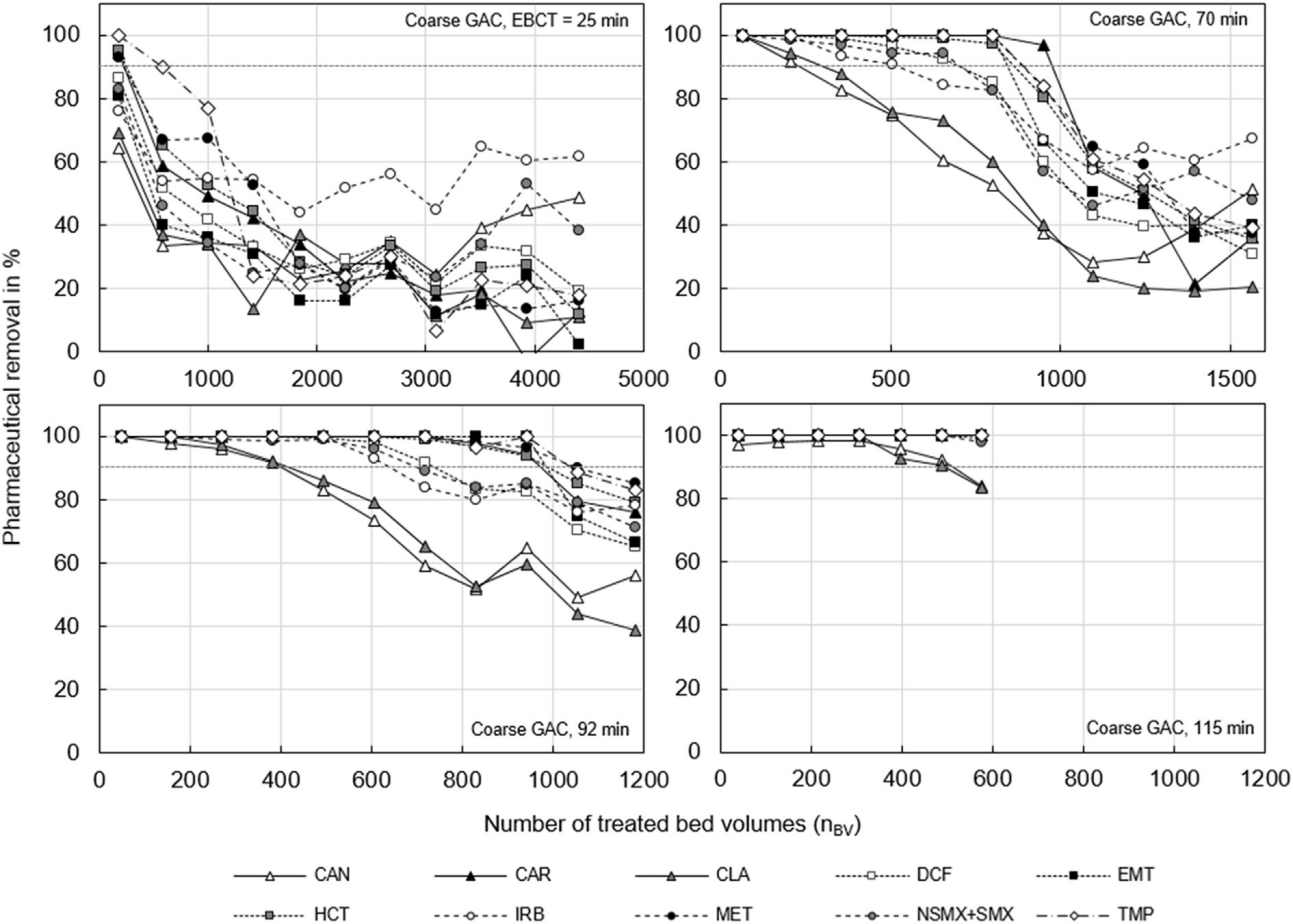
Removal of pharmaceuticals with coarse GAC as a function of the number of treated bed volumes (n_BV_) for empty bed contact times (EBCT) of 25, 70, 92 and 115 min.

**Fig. 2:**
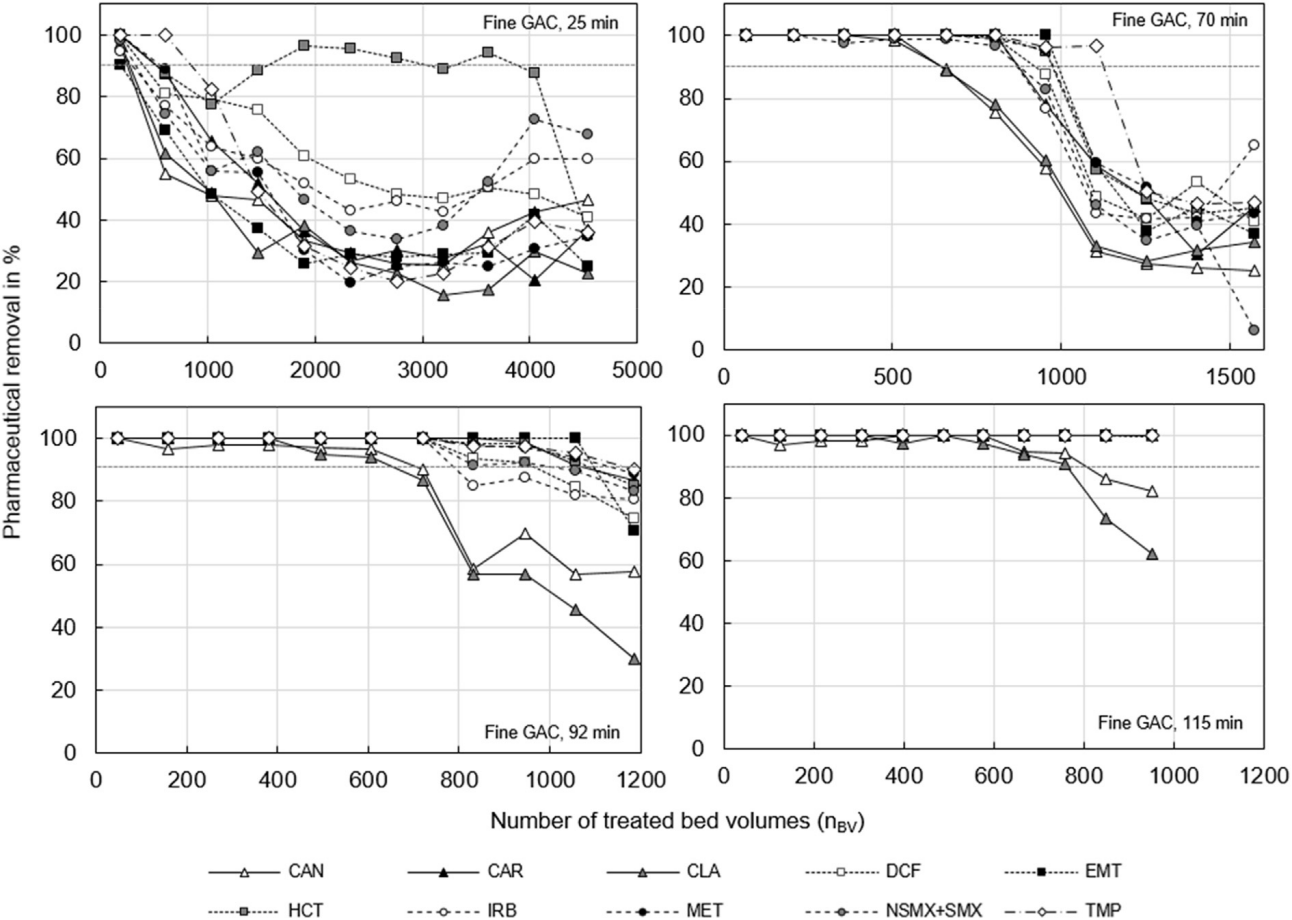
Removal of pharmaceuticals with fine GAC as a function of the number of bed treated volumes (n_BV_) for empty bed contact times (EBCT) of 25, 70, 92 and 115 min.

For all compounds treated with coarse and fine GAC, immediate breakthrough occurred at EBCTs of 25 min (Fig. 1 and Fig. 2, respectively). Based on our results, we suggest a minimum EBCT of 70 min. Longer EBCTs only result in a small increase of the n_BV_ until breakthrough. Especially the removal of CAN and CLA, the two least adsorbing compounds, did hardly increase when the contact times were longer than 70 min.

Although the suggested EBCT of at least 70 min is long, it is not critical, because the volume of the GAC column is small compared to the volume of the main treatment step, which is the nitrification reactor. Assuming that the average retention in the nitrification reactor is approximately 3 days (Fumasoli et al., 2016) and assuming an EBCT of 70 min for the GAC column, the required volume for the GAC column would be 60 times smaller than for the nitrification reactor. This means that the necessary space for the GAC treatment is small. Additional experiments with sampling at EBCTs between 25 and 70 min could show, whether shorter EBCTs than 70 min can also achieve satisfactory removal.

The recommended EBCT for urine treatment is much longer than what is suggested for the treatment of WWTP effluents. Typical EBCTs tested for advanced wastewater treatment range between 15 and 25 min (Altmann et al., 2016b; Kårelid et al., 2017; and Bourgin et al., 2018). However, ongoing studies on pharmaceutical removal suggest that EBCTs at the higher edge, that is 25 min, are better for an efficient pharmaceutical removal from WWTP effluents (Wunderlin et al., 2017).

The fast breakthrough could have been caused by the high content of natural organic matter (NOM). The influent of the nitrified urine had an average DOC concentration of 103 ± 20 mg/L (Table S 4, SI), while the typical DOC in the effluent of a wastewater treatment plant is about 5 mg/L (Table S 13, SI). The very high amount of NOM in the influent must have slowed down the adsorption of the pharmaceuticals due to at least two effects: first, by competing for adsorption sites and, second, by blocking pores and thereby slowing down surface diffusion (Worch, 2012). The high NOM concentrations could also explain why the removal of all pharmaceuticals did not drop to zerofor EBCT 25 min and 70 min (Fig. 1, Fig. 2). Biodegradation might have removed NOM, thereby freeing adsorption sites for pharmaceuticals (Worch, 2012). Biodegradation of pharmaceuticals could also have caused the persistent pharmaceutical removal in the GAC columns (Bourgin et al., 2018). However, this effect might only be of minor importance. Oezel Duygan and co-workers (in prep.) found that biodegradation of most pharmaceuticals is low in urine nitrification. When looking at pharmaceuticals, which were also used in this study, biodegradability was low, for DCF, EMT, HCT, SMX and TRI, while a high aerobic biodegradability was found for CLA.

### 3.2 Influence of GAC grain size

**Fig. 3:**
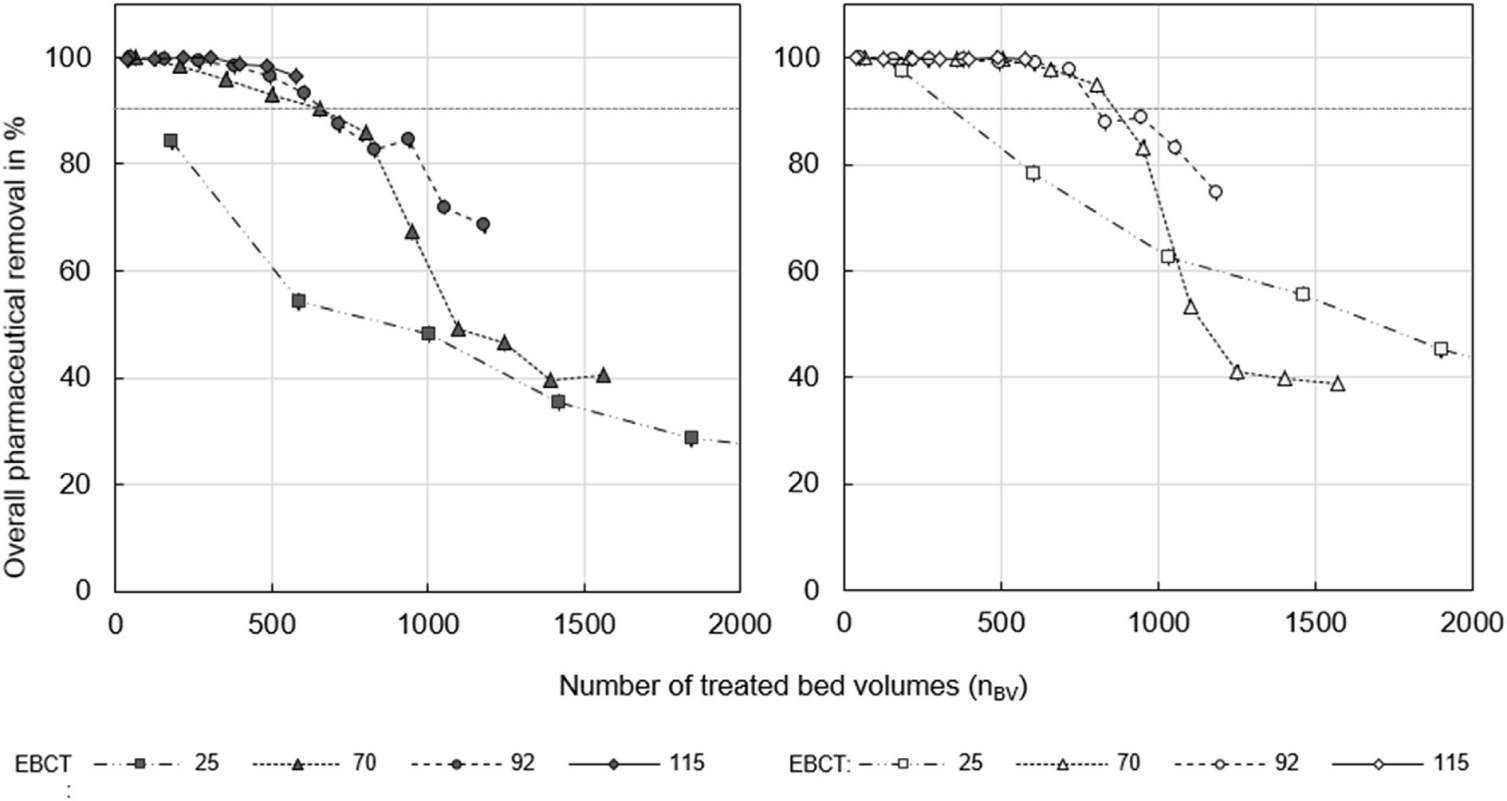
Overall removal of all pharmaceuticals with coarse (left) and fine (right) GAC as a function of the number of treated bed volumes (n_BV_) for empty bed contact times of 25, 70, 92 and 115 min.

Breakthrough occurred later with fine GAC than with coarse GAC (see Fig. 1, Fig. 2, and Figures S 11 to S 15). Especially for compounds with a lower tendency for adsorption (CAN and CLA) we achieved a better removal with fine GAC. For well adsorbing compounds, such as CAR, EMT, HCT, MET or TMP, the influence of the GAC grain size was not significant. Breakthrough of CAR, EMT, HCT and TMP occurred at the same time for coarse and fine GAC (at about n_BV_ = 700 for EBCT of 70 min). The difference in breakthrough is due to slower mass transfer in the larger particles of the coarse GAC. According to Worch (2012) the intraparticle mass transfer coefficient is reciprocally proportional to the radius of the GAC particle. Consequently, fine GAC can be expected to have steeper breakthrough curves with later onset compared to coarse GAC. Although breakthrough occurred earlier for coarse GAC, the measurement data show that the overall surface available for adsorption was probably similar for both grain sizes. The n_BV_ until inclination of the breakthrough curve is a measure for the total surface available for adsorption, because at this n_BV_ the activated carbon would be completely saturated under ideal conditions that means without limitation by adsorption kinetics (Worch, 2012). For fine and coarse GAC, the inclination points of the breakthrough curves were reached at about the same n_BV_ (e.g. n_BV_ = 1000 at EBCT of 70 min for overall pharmaceutical removal, see Fig. 3). The similarity of the available surface for adsorption makes sense, if the internal surface is dominating adsorption. While the external surface of the fine and coarse GAC differed by a factor of 2.5 (see section S 7.4, SI), the total mass of GAC and thereby the internal surface in the two columns were about the same (see 2.1 Experimental setup). The internal surface of GAC can be assumed to be directly proportional to the mass, because ball milling and sieving does not change the specific internal surface (Aumeier et al., 2019).

### 3.3 Overall removal of pharmaceuticals

The positive effect of fine GAC on the elimination is clearly visible for the overall removal of all eleven tested pharmaceuticals (see Fig. 3, Table S 11 and Table S 12, SI). For all EBCTs, breakthrough occurred earlier when coarse GAC was used and maximally n_BV_ = 487 could be treated with the longest EBCT of 115 min. If fine GAC was used, n_BV_ until breakthrough at the longest EBCT was almost doubled (n_BV_ = 758). For the suggested minimum EBCT of 70 min, breakthrough occurred at n_BV_ = 209 for coarse and at n_BV_ = 659 for fine GAC. The total number of treated bed volumes is determined by CAN and CLA for which we obtained significantly lower removal and fast breakthrough (see section 3.1). If the removal between the single compounds vary widely, the median might be more representative for the overall removal than the average. In our case, this would increase the time until breakthrough from n_BV_ = 209 to 504 for coarse GAC at EBCT of 70 min. The overall removal for coarse and fine GAC at the shortest EBCT of 25 min is shown in Fig. S 15 (SI).

### 3.4 Comparison with pharmaceutical removal from municipal wastewater

Removal of pharmaceuticals is more efficient for nitrified urine than for WWTP effluent according to a comparison of the CURs. To compare the treatment efficiency in our experiment with literature data, we used the data for the column with fine GAC, at an EBCT of 92 min and a removal of 90%. The average CUR, which corresponds to the amount of carbon required to treat 1 L of nitrified urine, was 569 mg/L (Table S 18). The average n_BV_ was calculated to be 1040 (Table S 15). In a study on a German WWTP, Altmann et al. (2016b) reported CURs between 20 and 30 mg GAC/L and n_BV_ until breakthrough of 9.000–14.000 for well-adsorbing compounds, such as CAR, DCF and MET, and for a treatment goal of 80%. Reported CURs are significantly higher for higher treatment goals. Swedish researchers reported an average CUR of 110 mg/L to achieve a 95% removal of pharmaceuticals from WWTP effluent (Kårelid et al., 2017). For an average removal of organic micropollutants by 80% in Swiss wastewaters, a CUR of 10–20 mg PAC/L was estimated to be sufficient (Siegrist et al., 2019). To evaluate our results with the adsorptive removal of pharmaceuticals from WWTP effluent, we used datasets for the treatment of Swiss WWTP effluents from Wunderlin et al. (2017) and Bourgin et al. (2018) and calculated n_BV_ and CURs for a removal of ≥90%. General information on the influent characteristics and the GAC treatment can be found in Table S 13 (SI). Influent concentrations of pharmaceuticals are compiled in Table S 14 (SI) and results of our calculations in Table S 15 to S 20 (SI). Our calculations showed that the average CURs to remove 90% of pharmaceuticals from Swiss WWTP effluent range between 95 and 160 mg GAC/L (Table S 18, SI) and the average n_BV_ until breakthrough range between 5800 and 7620 (Table S 15, SI). These CURs are much lower than for urine, and the n_BV_ until breakthrough are substantially higher, indicating that the pharmaceutical removal from 1 L municipal WWTP effluent is more efficient. If the CUR is referred to the DOC mass and not to the liquid volume, an average CUR of 5.5 mg GAC/mg DOC was required for urine treatment, which is substantially lower than the CURs of 18–29 mg GAC/mg DOC for municipal wastewater (Table S 19, SI). However, it is possible that the nature of the DOC in nitrified urine and thus its affinity to GAC is substantially different to the DOC in WWTP effluent. Furthermore, the DOC concentration in WWTP effluent is much lower (about 5.4 mg C/L, Table S 13), than in nitrified urine (103 mg C/L, Table S 13). In order to consider the higher concentrations of pharmaceuticals and DOC in nitrified urine compared to WWTP effluent, CUR can be converted to the mass of GAC required per person and day assuming a urine flow of 1.25 L/(p·d) (Udert et al., 2006) and a wastewater flow of 350 L/(p·d) (Gujer, 2007). With this assumptions, we obtain an average GAC demand per person and day of 0.71 g/(p·d) for nitrified urine and of 33–56 g/(p·d) for WWTP effluent (Section 8.1, SI). The GAC demand for pharmaceutical removal in this example is about 60 times or more than one order of magnitude smaller for urine treatment than for the treatment of WWTP effluent. This calculation is based on the assumption that the pharmaceuticals excreted with feces will not contribute substantially to the concentration of dissolved pharmaceuticals in WWTP effluents. Oezel Duygan et al. (in prep.) did the same calculation for treatment of nitrified urine with PAC, and found that at least 10 times less PAC is needed compared to treat municipal wastewater.

### 3.5 Degradation of pharmaceuticals in the influent tank

Until the end of the experiments, the influent concentration of EMT, HCT and NSMX + SMX decreased by more than 20% (Fig. S 9, SI). Final degradations of 64% for EMT (from 2.57 to 0.92 μg/L), 62% for HCT (from 84.5 to 32.1 μg/L) and 36% for SMX + NSMX (from 11.4 to 7.3 μg/L) were observed. The influent tank was a standard intermediate bulk container (IBC) with no specific protection against light or surrounding air. The degradation of these compounds might be a combination of biological and chemical processes. Biofilm growth on the inside of the container walls was observed at the end of the experiment, which indicates biological activity inside the container. High removal of HCT during storage has been reported but only for stored fresh urine at solution pH of 9 (Oezel Duygan et al. in prep.). Further investigations would be necessary to understand pharmaceutical degradation in stored nitrified urine.

### 3.6 Influence of GAC treatment on pH and nutrient concentrations

The solution pH and the concentrations of nutrients like ammonia, nitrate, phosphate, potassium, sulfate and other urine compounds, such as chloride and sodium, are not affected by the GAC treatment for relevant EBCTs (>25 min). The overall removal of the urine nutrients and trace compounds in both columns ranges between −5% and +3% (Fig. S16 and SI) and the absolute difference between influent and effluent pH was 0.05 pH units for the coarse and 0.06 pH units for the fine GAC (Fig. S6 and SI). The changes of nutrients are insignificant considering that the typical standard deviation of the chemical measurements used in our study is 5%. These insignificant changes allow the conclusion that no nutrients are lost when pharmaceuticals are removed from nitrified urine by adsorption on GAC. In contrast, phosphorus and nitrogen removal of up to 36% from synthetic fresh urine using different biochars where reported by Solanki and Boyer (2017) and Tarpeh and co-workers actually used biochar to recover ammonia from stored urine for fertilizer production (Tarpeh et al., 2017). The high nutrient removal in these two studies could be due to the different chemical speciation in fresh and stored urine and the high pH value in stored urine (around pH 9). Another reason could be differences in carbon properties. Our results are in accordance with the study by Oezel Duygan et al. (in prep.), who studied the treatment of nitrified urine with PAC for pharmaceutical removal. Oezel Duygan and co-workers observed high removal of pharmaceuticals but no removal of urine nutrients.

For fine GAC we observed a local anomaly of the phosphate concentration at the sampling port for EBCT = 25 min. At this point, phosphate was removed on average by almost 25% (Figure S 17, left, SI). The removal was not constant over time but increased continuously with the duration of the experiment. The phosphate concentrations correlated with the pH values (Figure S 17, right, SI). In addition to the drop of the pH value and of the phosphate concentration, we observed white stains in the GAC bed around the sampling port for EBCT = 25 min (Figure S 18, SI) accompanied by a reduced flow rate during sampling, most probably caused by precipitated minerals inside the sampling tube. The observations we made at the sampling point for EBCT = 25 min, were most probably due to nitrification by acid-tolerant ammonium oxidizing bacteria (Fumasoli et al., 2017), leading to brass corrosion and local precipitation of metal phosphates (see section 9, SI for a more detailed discussion). Brass corrosion is also known as dezincification (Tuck et al., 2010), which can release metal ions, such as Fe, Al, Cu, and Zn, dependent on the material composition. To test the proposed mechanism, we executed batch experiments with the corroded sampling port (see section S 9.1, SI). We found, that the phosphate concentration decreased by about 12% if the pH decreased from 6.45, which is typical for nitrified urine, to 5.0 (Figure S 19, SI). ICP-OES analysis of the digested precipitates revealed that the solids contained high concentrations of zinc (63 g/kg), intermediate amounts of Cu (3.6 g/kg), Fe (1.45 g/kg) and Ni (1.68 g/kg), and trace amounts of Pb (0.96 g/kg) (Figure S 20, SI). The measurements support our hypothesis that the release of metals by corrosion of brass caused the local drop in phosphate concentration and that the locally observed phosphate removal is is caused by corrosion and precipitation and not by adsorption on activated carbon.

### 3.7 Using UV absorbance or DOC removal to predict overall removal of pharmaceuticals

**Fig. 4:**
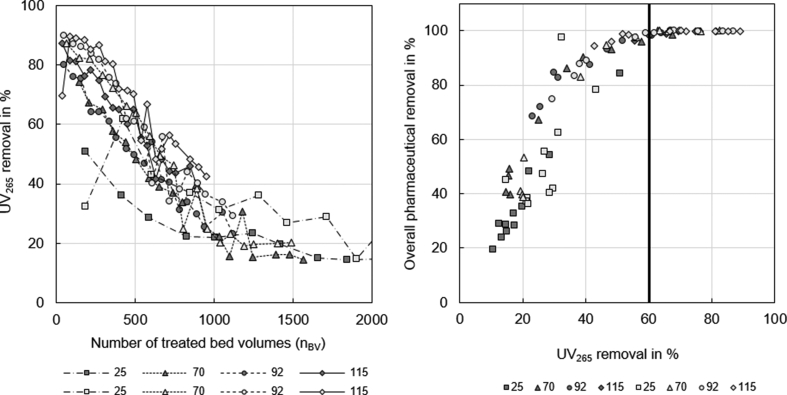
UV_265_ removal as a function of the number of treated bed volumes (left) and overall removal of all pharmaceuticals as a function of UV_265_ removal (right) at EBCTs of 25, 70, 92 and 115 min for the adsorption on coarse (dark grey symbols) and fine (light grey symbols) GAC.

UV absorbance was measured at a wavelength of 265 nm, because at the more commonly used wavelength of 254 nm, strong interference with nitrate was observed (section 10, SI). The influent UV_265_ of 0.31 ± 0.07 AU was reduced by the treatment with coarse and fine GAC for EBCTs longer than 25 min. UV_265_ removal was highest for the longest EBCT (115 min) and fine GAC, although the difference to coarse GAC and all other EBCTs was small (Fig. 4, left). Results are similar for DOC removal, although the DOC drops already at the beginning of the experiments to about 80% (Figure S 21, SI). High UV_265_ removal was an indication for high overall pharmaceutical removal. Up to a UV_265_ removal of 60% overall pharmaceutical removal increased steadily, while at higher UV_265_ removal, the overall pharmaceutical removal was already about 100% (Fig. 4, right). UV_265_ removal and overall pharmaceutical removal (Fig. 4, right) showed a better correlation than DOC removal and overall pharmaceutical removal (Fig. S 22, left, SI). Nevertheless, both parameters are suitable to give a reliable threshold value above which overall pharmaceutical removal is 100%. Similar threshold values for treatment of WWTP effluents with activated carbon can be found in the literature. For PAC and well-adsorbing compounds, such as CAR and MET, it was reported that a UV_254_ decrease of around 25% indicate a pharmaceutical removal of over 80%, whereas a UV_254_ decrease of over 50% is necessary for 80% removal of weakly adsorbing pharmaceuticals, e.g. ofiomeprol and primidone (Altmann et al. 2016a). In our study, we observed the similar correlations for UV_265 removal._ For a pharmaceutical removal of more than 80% (when excluding the measurements at the lowest EBCT of 25 min) a UV_265_ removal of at least 25% is necessary for well adsorbing compounds (e.g. HCT, TMP), while for the weakly adsorbing compounds CLA and CAN, a UV_265_ removal of 50% is needed (Fig. S 23, SI). A UV_265_ removal of 40% corresponds to an average pharmaceutical removal in the range of 80–90%. Our experiments therefore confirm the statement of Altmann and co-workers, who postulated that the correlation of UV absorbance removal and pharmaceutical removal is only minimally affected by the wastewater composition.

We conclude that measuring UV_265_ absorbance allows for real-time monitoring and control of the flow-through columns. However, it has to be taken into account that in the case of nitrified urine the samples need to be diluted beforehand and corrected for blanks and interferences by nitrate.

## 4 Conclusion

We could verify the hypothesis, that adsorption on GAC in a flow-through filter removes pharmaceuticals from nitrified urine without losing significant amounts of nutrients.

i. All eleven pharmaceuticals, including compounds with high and low tendencies for adsorption on activated carbon, were removed. The earliest breakthrough was calculated for candesartan (CAN) and clarithromycin (CLA).
ii. Complete pharmaceutical removal was achieved for up to 660 bed volumes at an empty bed contact time (EBCT) of 70 min. Longer EBCTs only slightly increased n_BV_ until breakthrough. At an EBCT of 25 min, the removal was insufficient, probably due to strong competition with natural organic matter (NOM). EBCTs shorter than 70 min might be sufficient, but need to be tested, preferably in pilot studies.
iii. With fine GAC, breakthrough occurred later, probably due to the shorter intraparticle diffusive path. Consequently, less GAC is required to treat the same urine volume when using fine GAC.
iv. Nearly two orders of magnitude less activated carbon would be needed per person equivalent, if pharmaceuticals were removed on site from nitrified urine instead of removing them from the effluent of a centralized WWTP.
v. Nutrient removal is negligible in GAC treatment of nitrified urine. A local drop in phosphate concentration was an experimental artefact due to the corrosion of a brass valve triggered by acidophilic nitrification.
vi. DOC and UV_265_ measurements can provide threshold values indicating complete pharmaceutical removal. Due to the high concentrations in nitrified urine, pre-dilution might be necessary for online UV_265_ measurements.

## Declaration of competing interest

The authors declare that they have no known competing financial interests or personal relationships that could have influenced the work reported in this paper.
