# Supplementary material for: Removal of pharmaceuticals from nitrified urine by adsorption on granular activated carbon
Source: Water Res X. 2020 Jun 2;9:100057. doi: 10.1016/j.wroa.2020.100057 (PMC7298675; doi:10.1016/j.wroa.2020.100057)
Supplement: Multimedia component 1 [file mmc1.pdf]

Supporting Information for

Removal of pharmaceuticals from nitrified urine by adsorption on granular activated carbon

Isabell Köpping[a], Christa S. McArdell[a], Ewa Borowska[a,1], Marc A. Böhler[a] and Kai M. Udert[a, b, *]

[a] Eawag, Swiss Federal Institute of Aquatic Science and Technology, 8600 Dübendorf, Switzerland

[b] ETH Zürich, Institute of Environmental Engineering, 8093 Zürich, Switzerland

Water Research X (2020)

https://doi.org/10.1016/j.wroa.2020.100057

[*] Corresponding author: Kai M. Udert, Kai.Udert@eawag.ch

Eawag, Swiss Federal Institute of Aquatic Science and Technology,

Department of Process Engineering,

8600 Dübendorf, Switzerland

[1] Current address: Karlsruhe Institute of Technology (KIT), Engler-Bunte-Institut, Water Chemistry and Water Technology, DE-76131, Karlsruhe, Germany

List of contents

List of contents 2

S 1 Properties of the activated carbon 3

S 2 Procedures for pharmaceuticals 4

S 2.1 Calculation of pharmaceuticals in reference urine 4

S 2.2 Preparation of spiking solution 4

S 3 Experimental setup 5

S 3.1 Investigated empty bed contact times 5

S 3.2 Calculation of Reynolds number 5

S 4 Pharmaceutical analysis 8

S 4.1 Calculation of pharmaceuticals in reference urine 8

S 4.2 Preparation of spiking solution 8

S 4.3 Pharmaceutical analysis 8

S 4.4 Preparation of citrate buffer 10

S 4.5 Determination of limit of quantification 10

S 4.6 Determination of relative recoveries 11

S 5 UV absorbance measurements in nitrified urine 12

S 6 Operation of the GAC columns 13

S 7 Removal of pharmaceuticals during GAC treatment 17

S 7.1 Pharmaceutical degradation in influent tank 17

S 7.2 Pharmaceutical removal during treatment with GAC 17

S 7.3 Overall removal of investigated pharmaceuticals 20

S 7.4 Calculation of specific surface 21

S 8 Comparison with advanced wastewater treatment 23

S 8.1 Calculation of personal CUR 26

S 8.2 Influence of urine nutrients by GAC treatment 27

S 9 Local removal of phosphate 28

S 9.1 Batch experiments to investigate the fate of dissolved phosphate 29

S 9.2 Experimental procedure 29

S 9.2.1 Solid analysis 29

S 9.2.2 Results 29

S 10 UV_{265} removal and DOC removal as surrogate parameter for pharmaceutical removal 31

References 33

S 1 Properties of the activated carbon

In this section additional information about the activated carbon used in this study is given.

Table S 1 Properties of the granular activated carbon

	Column 1	Column 2
Name	Norit® GCN 830	Norit® GCN 830
Company	Norit, AC Amersfoort, Netherlands	Norit, AC Amersfoort, Netherlands
Raw material	Coconut shell	Coconut shell
Range grain size*	1.4 - 2.4 mm	0.6 - 1.0 mm
	Mesh 14 x 8	Mesh 30 x 18
Bed density	0.53 g/cm^3	0.58 g/cm^3
Bed porosity	0.43	0.39
Information about the original material given by the manufacturer		
Median particle size	1.68 mm	
Iodine number	925	
Nitrogen iso BET	982 m^2/g	

* The fractionation of the GAC was done by sieving the original material with standard sieves.

S 2 Procedures for pharmaceuticals

In this section further information of the preparation, spiking, analysis and the evaluation of the selected pharmaceuticals is given.

S 2.1 Calculation of pharmaceuticals in reference urine

Data on the average pharmaceutical concentrations in biologically treated municipal wastewater was obtained from seven WWTPs in Switzerland and Germany (Götz et al. 2014). The values of relative excretion e_{urine} were calculated by dividing the excretion rates for urine (E_{urine}) with the sum of excretion via urine (E_{urine}) and feces (E_{feces}) (Equation 2), assuming that all pharmaceuticals in municipal wastewater originated from both urine and feces.

$$c_{u,ref} = c_{WW,bio} \cdot e_{urine} \cdot 100 \quad (1)$$

$$e_{urine} = \frac{E_{urine}}{E_{urine} + E_{feces}} \quad (2)$$

S 2.2 Preparation of spiking solution

A mixture containing all compounds was prepared by dissolving the necessary amounts of all analytes in about 30 mL of methanol. To reduce the organic carbon content originating from the solvent that might interfere with the removal efficiency, the solvent of the pharmaceutical mixture was evaporated at 35 °C in a N_2-airstream by a factor of two. To ensure a good mixing, the pharmaceutical-methanol mix (15 mL) was subsequently added to increasing volumes of nitrified urine (500 mL → 5 L → 80 L). Finally, a second container filled with about 1100 liter of nitrified urine was connected and the spiked nitrified urine was circulated during five hours with a drum pump.

For nitrified urine with a normal background DOC of around 100 mg/L, the addition of the pharmaceutical mix is not substantially affecting the influent DOC concentration. Actually, the DOC increase due to spiking change is within the measurement accuracy of the selected analytical method.

S 3 Experimental setup

In this section additional information related to the experimental setup and sampling procedure are given.

S 3.1 Investigated empty bed contact times

When planning the experiment, we wanted to investigate five EBCTs, the longest with a GAC bed height of 50 cm, corresponding to an EBCT of about 230 min. During the total operation time of 74 days, we did not observe breakthrough of any compound at this sampling point. Therefore, we did not include the results for sampling ports H1.5 and H2.5.

S 3.2 Calculation of Reynolds number

Crittenden et al. (2012, p. 1242) recommends to keep the Reynolds number (Re) greater than 0.1 for synthetic organic chemicals in small GAC columns. Re above 0.1 ensures that axial dispersion is not limiting mass transfer. In small columns, axial dispersion is caused by molecular diffusion (Crittenden et al., 2012). We obtained Re 0.08 for the fine material and 0.17 for the coarse material by using Equation 3 and parameter values:

$$Re = \frac{\rho_l \cdot v_s \cdot d}{\varepsilon \cdot \mu} \tag{3}$$

Re	Reynolds number
ρ_l	density of liquid
d	particle diameter of adsorbent
v_s	superficial velocity
ε	bed porosity
μ	dynamic viscosity

d =	1.9 mm (coarse material, approximated)	
d =	0.8 mm (fine material, approximated)	
ε =	0.43	(coarse material)
ε =	0.39	(fine material)
v_s =	0.14 m/h	
ρ_l =	1000 g/L	
μ =	1 g/m/s	

Table S 2 Sampling points of each GAC column and the corresponding bed volumes (V_b), empty bed contact times (EBCT) and GAC mass

Sampling points	Grain size	GAC bed height	V_b	EBCT*	GAC mass
		[cm]	[mL]	[min]	[g]
H1.1	Coarse	5.5	124	25	66
H1.2	Coarse	15.5	350	70	185
H1.3	Coarse	20.5	463	92	245
H1.4	Coarse	25.5	575	115	305
Outflow 1	Coarse	64.5	1455	290	771
H2.1	Fine	5.3	120	24	70
H2.2	Fine	15.3	345	68	201
H2.3	Fine	20.3	458	91	267
H2.4	Fine	25.3	571	113	333
Outflow 2	Fine	64.3	1451	287	846

* To simplify the presentation of the results, we used the EBCTs of 25, 70, 92 and 115 min for both columns.

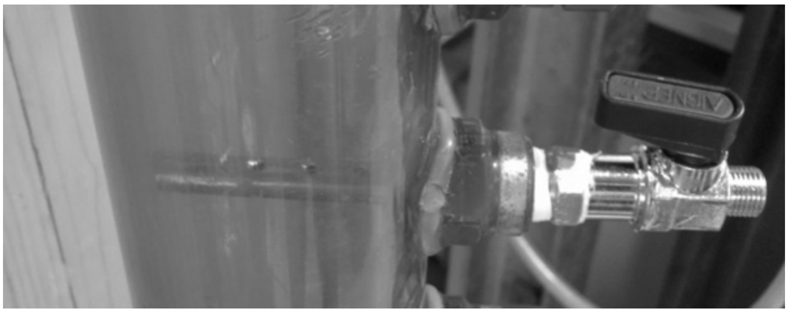

Figure S 1 Construction of sampling port with perforated sampling tube

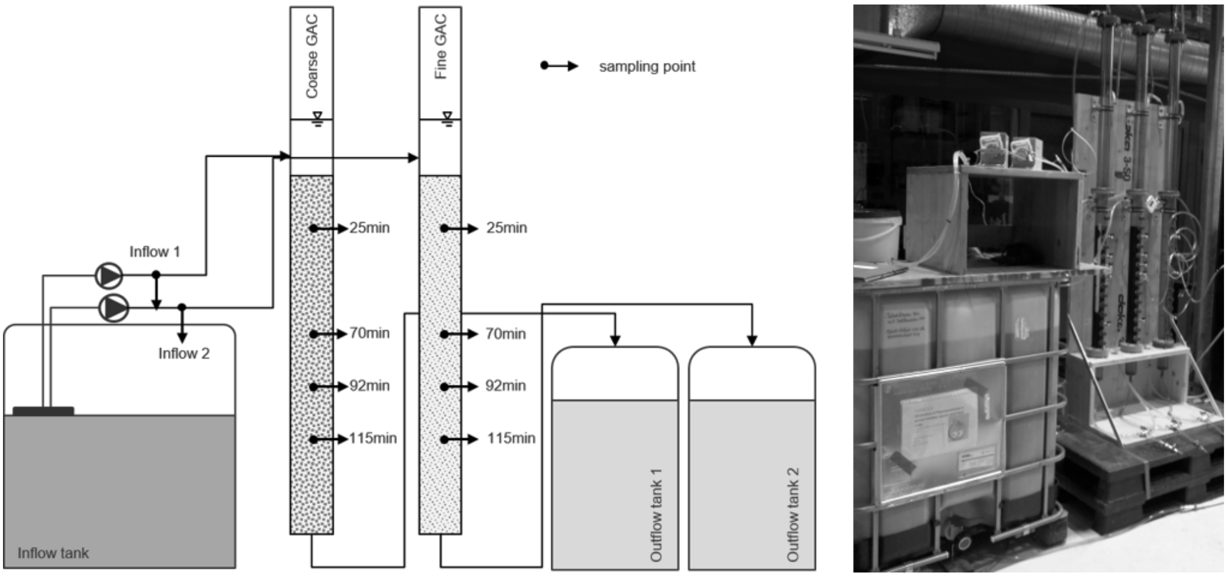

Figure S 2 Process scheme and picture of experimental setup

Table S 3 List of measured parameters

Parameter	Code	Unit	Measured in…
Temperature	T	[°C]	Influent 1+2, effluent 1+2
Conductivity	C	[mS/cm]	Influent 1+2, effluent 1+2
pH value	pH	[-]	Influent 1+2, effluent 1+2, after 30 days as well H1.1, H1.2, H1.3, H1.4, H2.1, 2.2, H2.3, H2.4
Dissolved organic carbon	DOC	[mg/L]	All sampling points
UV absorbance at 265 nm	UV_{265}	[AU] "absorbance unit"	All sampling points
Nitrate, ammonia, phosphate, Potassium, sulfate, sodium, Chloride, calcium, magnesium	NO_3-N, NH_4-N, PO_4-P, K, SO_4, Na, Cl, Ca, Mg	[mg/L]	All sampling points
Candesartan, Carbamazepine, Clarithromycin, Diclofenac, Emtricitabine, Hydrochlorothiazide, Irbesartan, Metoprolol, Sulfamethoxazole, N_4-acetylsulfamethoxazole, Trimethoprim	CAN, CAR, CLA, DCF, EMT, HCT, IRB, MET, SMX, NSMX, TMP	[µg/L]	Influent 1+2 and H1.1, H1.2, H1.3, H1.4, H2.1, H2.2, 2.3, H2.4

Table S 4 Properties and average concentrations in nitrified urine used in this study given as average ± standard deviation (n=21)

Parameter	Unit	Nitrified urine
pH	[-]	6.9 ± 0.3
DOC	[mg/L]	103 ± 20
NH_4^+	[mg N/L]	2110 ± 60
NO_3^-	[mg N/L]	2080 ± 40
PO_4^{3-}	[mg P/L]	199 ± 10
Ca	[mg/L]	25 ± 5
Cl	[mg/L]	2890 ± 60
K	[mg/L]	1450 ± 190
SO_4^{2-}	[mg /L]	745 ± 22
Na	[mg/L]	1680 ± 70

S 4 Pharmaceutical analysis

In this section further information on the preparation, spiking, analysis and the evaluation of the selected pharmaceuticals is given.

S 4.1 Calculation of pharmaceuticals in reference urine

Data on the average pharmaceutical concentrations in biologically treated municipal wastewater were obtained from seven WWTPs in Switzerland and Germany (Götz et al. 2014). The values of relative excretion e_{urine} were calculated by dividing the excretion rates for urine (E_{urine}) by the sum of excretion via urine (E_{urine}) and feces (E_{feces}) (Equation 5), assuming that all pharmaceuticals in municipal wastewater originate from both urine and feces.

$$c_{u,ref} = c_{WW,bio} \cdot e_{urine} \cdot 100 \quad (4)$$

$$e_{urine} = \frac{E_{urine}}{E_{urine} + E_{feces}} \quad (5)$$

S 4.2 Preparation of spiking solution

A mixture containing all compounds was prepared by dissolving the necessary amounts of all analytes in approximately 30 mL of methanol. To reduce the organic carbon content originating from the solvent that might interfere with the removal efficiency, the solvent of the pharmaceutical mixture was evaporated at 35 °C in a N_2-airstream by a factor of two. To ensure a good mixing, the pharmaceutical-methanol mix (15 mL) was subsequently added to increasing volumes of nitrified urine (500 mL → 5 L → 80 L). Finally, a second container filled with about 1100 L of nitrified urine was connected and the spiked nitrified urine was circulated during five hours with a drum pump. The concentrated pharmaceutical mix in methanol added about 5 mg/L in DOC to the urine sample, which was small compared to the DOC of around 100 mg/L for nitrified urine.

S 4.3 Pharmaceutical analysis

Shortly before analysis, samples were thawed and diluted 100 times with Nanopure® water to minimize matrix effects. After dilution, samples were spiked with isotope-labeled internal standards (Table S 5) to reach a concentration of 200 ng/L. Subsequently, the diluted samples were filtered through glass microfiber filters (GF/F, pore size 0.7 µm and on top: GF/D, pore size 2.7 µm, Whatman, Maidstone, United Kingdom). Before injection of the diluted urine sample (20 mL) into the online SPE system, the pH was stabilized by automatic addition of 80 µL of a 0.5 M citrate buffer solution. SPE cartridges used for enrichment contained Oasis® HLB sorbent (8-9 mg, 15 µm, Waters, USA) as first material and a mixture (9-10 mg) of anion exchanger Strata X-AW, cation exchanger Strata X-CW (30 µm, Phenomenex, UK) and Env+ (Biotage, Sweden) in a ratio of 1:1:1.5 (X-AW:XCW:ENV+) as second material. The cartridge was eluted with acetonitrile and ammonium acetate (2 mM) using the elution program presented in Table S 6. Separation of micropollutants was achieved with an Atlantis®T3 (3.0 x

150 mm, particle size 3 µm Waters, USA) HPLC column. For elution a gradient program, using MeOH and Nanopure® water (NPW), both acidified with 0.1% formic acid, was used according to Table S 7. MS data were acquired by a ThermoScientific™ Q-Exactive Plus™ high-resolution mass spectrometer. MS data were collected in full scan mode (100-900 m/z) at 70'000 resolution, using separately positive and negative electrospray ionization. Data were analyzed with Xcalibur™ (Thermo Scientific™, Switzerland) in the Qual Browser and Trace Finder 3.3 (Thermo Scientific™, Switzerland).

Table S 5 Investigated compounds, the corresponding internal standards and the ionization mode used for analysis

Analytes		Internal standard	Ionization mode
Candesartan	CAN	Candesartan-d5	Pos
Carbamazepine	CAR	Carbamazepine-d8	Pos
Clarithromycin	CLA	Clarithromycin-d3	Pos
Diclofenac	DCF	Diclofenac-d4	Pos
Emtricitabine	EMT	Emtrizitabine-13C, 15-N2	Pos
Hydrochlorothiazide	HCT	Hydrochlorothiazide-C13, d2	Neg
Irbesartan	IRB	Irbesartan-d3	Pos
Metoprolol	MET	Metoprolol-d7	Pos
N4-acetyl-sulfamethoxazole	NSMX	N4-Acetyl-Sulfamethoxazol-d5	Pos
Sulfamethoxazole	SMX	Sulfamethoxazole-d4	Pos
Trimethoprim	TMP	Trimethoprim-d9	Pos

Table S 6 Elution program for loading pump

Time [min]	2 mM Ammonium acetate [%]	Acetonitrile [%]	Flow rate [µL/min]
0	100		200
0.1		100	2000
0.6		100	2000
0.65	100		2000
5.6	100		2000
5.65	100		400
6.2	100		400
6.3		100	400
9.9		100	400
10.0	100		400
20.6	100		400
20.7	100		2000
32.0	100		2000
32.1	100		200

Table S 7 Elution program for the gradient pump

Time [min]	Methanol [%]	Water [%]	Flow rate [µL/min]
0	13	87	300
4	13	87	300
14	93	7	300
26	93	7	300
26.2	13	87	300
32.3	13	87	300

S 4.4 Preparation of citrate buffer

The citrate buffer was prepared by mixing a 0.5 M disodium hydrogen citrate solution (disodium hydrogen citrate sesquihydrate, Merck, in NPW) and a 0.5 M trisodium citrate solution (trisodium citrate dihydrate, Merck, in NPW) in the ratio 1:30 (v/v). The pH was adjusted to 7 by addition of a 1 M sodium hydroxide solution.

S 4.5 Determination of limit of quantification

The limit of quantification (LOQ) was determined once in NPW and once in urine. In NPW, the value of the carry-over was doubled and the calibration standard with the next higher concentration was multiplied by the dilution factor (100x) and taken as LOQ in NPW. In urine, LOQ was calculated as shown in Equations 6, 7 and 8. The carry-over is an average of the concentration of all blinds (with the exception of the first two blinds after the calibration curve).

$$Matrix\ factor = \frac{response\ ratio_{spiked\ sample} - response\ ratio_{sample}}{response\ ratio_{calibration\ standard}} \quad (6)$$

Where the response ratio is $\frac{area_{standard}}{area_{internal\ standard}}$ (7)

$$LOQ_{urine} = \frac{LOQ_{NPW}}{matrix\ factor} \quad (8)$$

The "spiked sample" was prepared by spiking 250 ng/L of the analyte to the sample (in the corresponding dilution). The matrix factor was determined separately for untreated urine (influent) and treated urine (effluent). The matrix factor was determined only during the second measurement slot in April 2016 but it was used also for the calculation of the LOQ of the influent urine in March 2016.

S 4.6 Determination of relative recoveries

Relative recoveries (RRs) were determined for untreated (influent) and treated (effluent) urine by firstly, subtracting the pharmaceutical concentration measured in the original sample (c_{sample}) from the pharmaceutical concentration measured in the spiked sample ($c_{spiked,\ measured}$) and secondly, by dividing the difference by the theoretical concentration of the spiked sample as shown in Equation 9. The spiked sample was prepared by spiking 250 ng/L of the analyte ($c_{spiked,\ theory}$) to the original sample considering the corresponding dilution.

$$RR\ [\%] = \frac{c_{spiked\ sample} - c_{sample}}{c_{spiked,theory}} \cdot 100\% \qquad (9)$$

Table S 8 **Measured influent concentrations (c_{inf}) at the start (t = 0 days) and the end (t = 74 days) of the experiment, limits of quantification (LOQ) obtained in three measurement campaigns (M1, M2 and M3) executed in February, April and November 2016 for influent and effluent samples, and relative recoveries (RR) for measurement campaigns M1 and M2.**

Compounds	c_{Inf}		LOQ influent		LOQ effluent			RR influent		RR effluent	
	t=0	t=74	M1	M2	M1	M2	M3	M1	M2	M1	M2
	[µg/L]		[µg/L]					[%]		[%]	
CAN	11.0	11.4	0.10	0.05	0.10	0.05	0.08	n.d.	106	69	97
CAR	5.4	5.5	0.05	0.05	0.05	0.05	0.04	78	112	82	110
CLA	51.9	45.6	0.50	1.00	0.50	1.00	0.50	n.d.	113	126	101
DCF	80.6	87.7	0.25	0.05	0.25	0.05	0.04	n.d.	119	82	99
EMT	2.6	0.9	0.25	0.05	0.25	0.05	0.18	68	103	70	96
HCT	84.5	32.1	0.05	0.05	0.05	0.05	0.04	n.d.	112	69	95
IRB	4.7	3.8	0.25	0.05	0.25	0.05	0.04	67	107	72	100
MET	27.0	27.1	0.50	0.05	0.50	0.05	0.10	n.d.	104	75	94
NSMX+SMX	11.4	5.7	0.10	0.05	0.10	0.05	0.52	68	108	71	97
TMP	4.6	4.6	0.25	0.05	0.10	0.05	0.08	67	108	72	98

n.d.: not determined

S 5 UV absorbance measurements in nitrified urine

Previous studies showed that the UV absorbance at 254 nm can be used to indicate the overall micropollutant removal performance of a wastewater treatment process see for example (Altmann et al., 2016; Kårelid et al., 2017; Zietzschmann et al., 2014). In this study, we investigated if this is also possible for the removal of pharmaceuticals from nitrified urine. For wastewater, typically, good correlation was found for the UV absorbance at 254 nm and the DOC or micropollutant concentration. Since nitrified urine is high in nitrate (concentration above 2000 mg/L) and we know that nitrate shows high absorbance in the UV/VIS range (Mašić et al., 2015), we were investigating if 254 nm can be used for nitrified urine as well. The absorbance spectra of an influent sample shows high absorbance for wavelengths ranging between 200 and 250 nm (Figure S 3, top, solid grey line). To identify the nitrate absorbance spectra, a concentrated nitrate solution (3000 mg/L) was prepared by dissolving potassium nitrate in nanopure water. The prepared nitrate solution showed strong absorbance in the range of 200 to 230 nm (Figure S 3, top, dotted black line). When the absorbance spectra of the influent sample is corrected by the blank (nanopure water) (Figure S 3 top, dashed black line) and the nitrate peak, a clear peak from 225 to 250 with its maximum at 236 nm (Figure S 3, top, solid black line) was observed. UV-Spectra of all samples were corrected in this manner. In the bottom of Figure S 3, the corrected spectra of samples taken after 56 days of operation and the treatment of 344 liter) after an EBCT of 25, 70, 91 and 115 minutes with coarse GAC are plotted. A clear decrease of the absorbance maximum and the absorbance between 250 and about 350 nm was observed for increasing EBCTs. Nevertheless, differentiation of the curves was best at a wavelength of 265 nm (minimum of curve H1.4 in the range of 250 and 300 nm) and was therefore selected to evaluate the UV/VIS absorbance of nitrified urine.

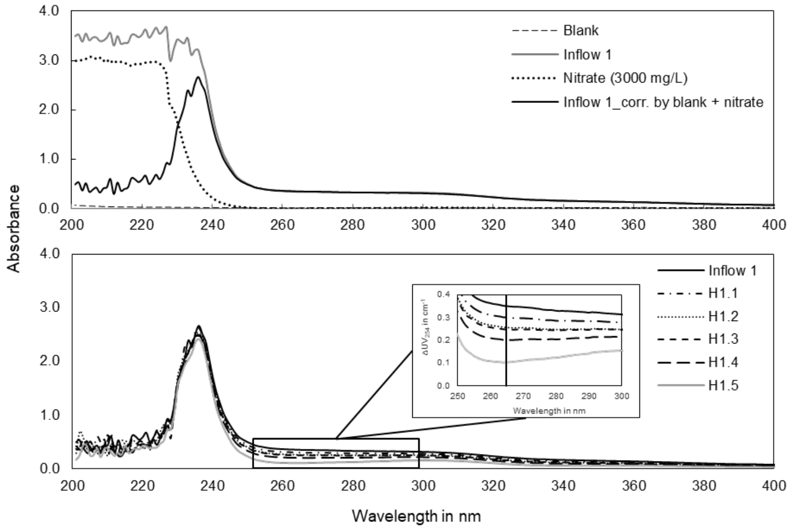

Figure S 3 UV absorbance measurements of Nanopure water (blank), untreated nitrified urine (inflow 1), a concentrated nitrate solution and the corrected absorbance spectra of an influent sample (top figure). UV absorbance spectra of samples from the influent and sampling points H1.1, H1.2, H1.3 and H1.4, corresponding to 25, 70, 92 and 115 minutes, of GAC column 1 (coarse GAC), all corrected by the blank and nitrate. All samples were taken at day 56 of the experiment (21.3.2016). The DOC influent concentration at this time was 113 mg/L and the DOC removal was 35, 29, 39 and 50 % for sampling points H1.1, H1.2, H1.3 and H1.4. Average overall removal of pharmaceuticals were 19, 49 and 84 % for sampling points H1.1, H1.2 and H1.3. Pharmaceutical removal at sampling port H1.4 was analyzed last on day 39 (3.3.2016) when close to 100 % removal was achieved.

S 6 Operation of the GAC columns

In the following section additional information on the operation of the GAC columns are given.

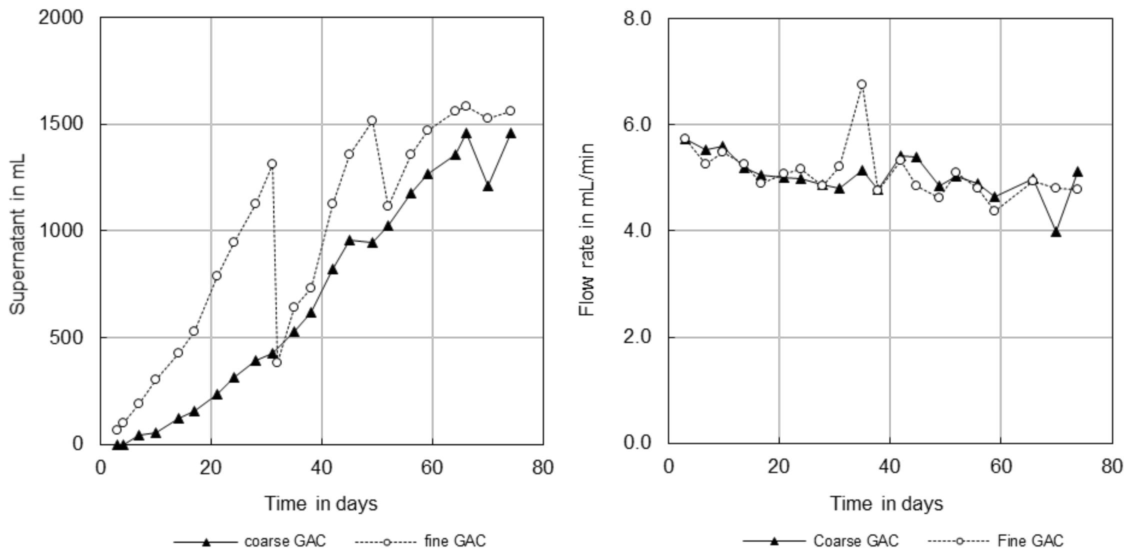

Figure S 4 Supernatant (left) and resulting flow rate (right) in GAC columns 1 and 2 over time

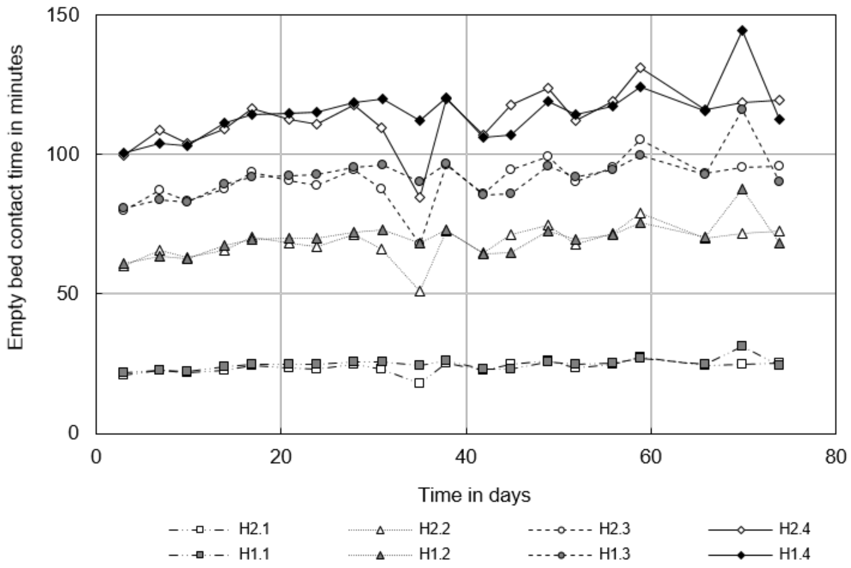

Figure S 5 Empty bed contact time as a function of time for all sampling points

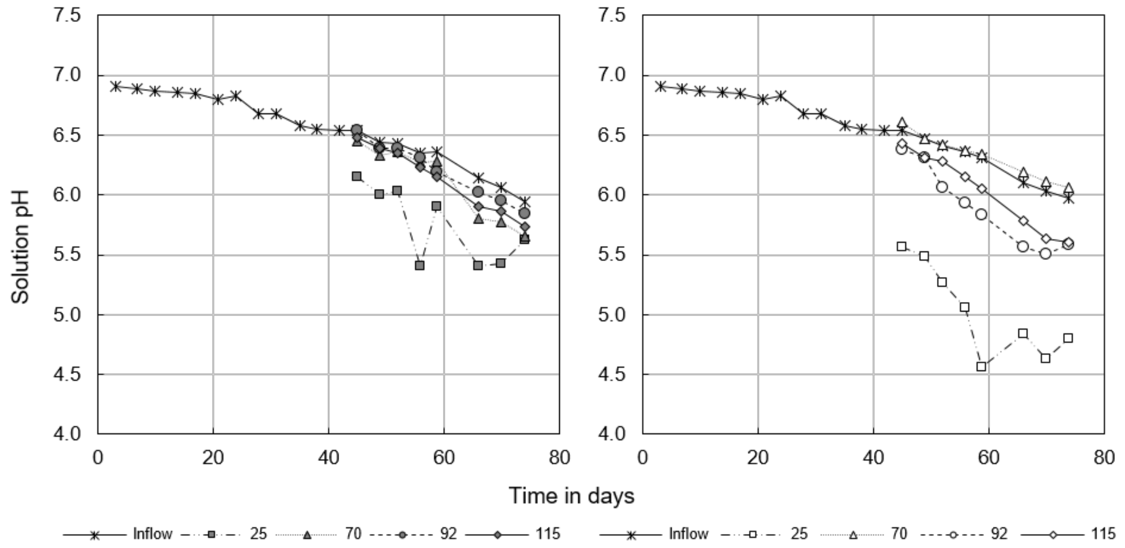

Figure S 6 **Solution pH measured in the influent and the effluent after empty bed contact times of 25, 70, 92 and 115 minutes treated with coarse (left) and fine (right) GAC**

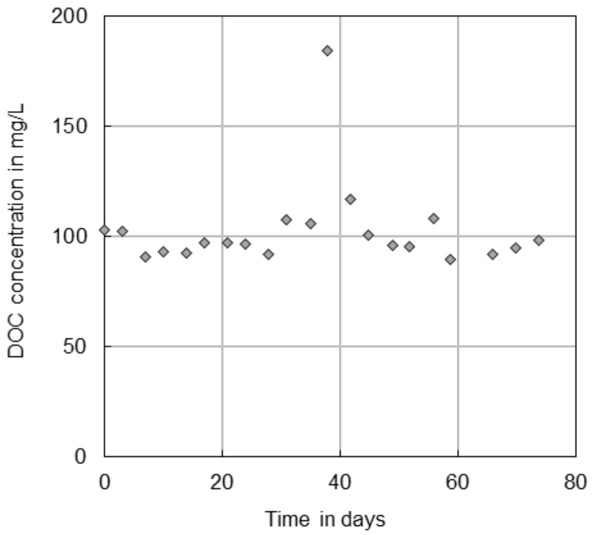

Figure S 7 **DOC concentration measured over time in the influent tank to the GAC columns.**

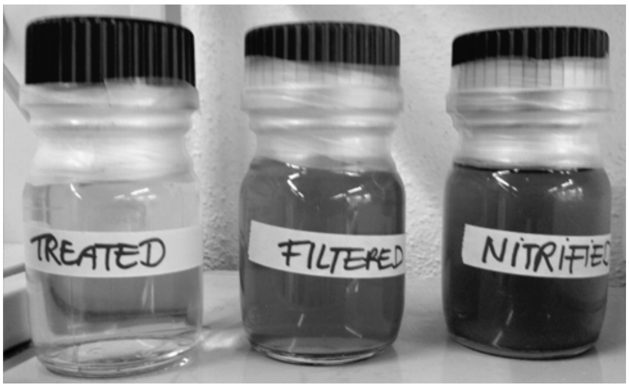

Figure S 8 **After the treatment with GAC the urine was almost colorless and odorless**

Table S 9 Operation parameters and concentrations measured over time in the effluent of sampling points H1.1, H1.2, H1.3 and H1.4 (coarse GAC). All values are rounded to three significant digits and given as average (AV) with standard deviation (SD), minimal and maximal values and the number of measured samples over time (n) used to calculate AV and SD.

		Pilot influents		H1.1 EBCT = 25 min	H1.2 EBCT = 70 min	H1.3 EBCT = 92 min	H1.4 EBCT = 115 min
	n	Av. ± SD (min - max)	n	Av. ± SD (min - max)	Av. ± SD (min - max)	Av. ± SD (min - max)	Av. ± SD (min - max)
T in °C	21	19.3 ± 0.4 (19 - 20)	8	20.8 ± 2 (19.5 - 25.8)	20.3 ± 0.8 (19.5 - 21.4)	20.4 ± 0.7 (19.5 - 21.4)	20.4 ± 0.8 (19.5 - 21.4)
pH	21	6.6 ± 0.3 (6.0 - 6.9)	8	5.7 ± 0.3 (5.4 - 6.2)	6.1 ± 0.3 (5.7 - 6.5)	6.2 ± 0.2 (5.8 - 6.5)	6.1 ± 0.3 (5.7 -6.5)
DOC in mg/L	21	103 ± 20 (90 - 185)	20	80.4 ± 21 (59.8 - 166)	67.3 ± 26.2 (29.4 - 152)	57.8 ± 24.4 (24.1 - 138)	46 ± 21 (21 - 118)
UV_{265} in AU	21	0.31 ± 0.07 (0.3 - 0.4)	20	0.26 ± 0.03 (0.16 - 0.29)	0.20 ± 0.06 (0.08 - 0.28)	0.16 ± 0.06 (0.07 - 0.25)	0.12 ± 0.05 (0.04 - 0.20)
Ca in mg/L	9	25 ± 5 (19 - 37)	8	21.5 ± 2.9 (17.8 - 25.5)	19.2 ± 8.4 (16.6 - 28.2)	20.3 ± 3.1 (15.6 - 26.6)	18.9 ± 4.3 (11.7 - 26.0)
Cl in mg/L	21	2890 ± 60 (2790 - 3030)	20	2930 ± 90 (2800 - 3190)	2910 ± 130 (2580 - 3110)	2940 ± 80 (2810 - 3090)	2930 ± 110 (2620 - 3210)
K in mg/L	21	1450 ± 190 (1330 - 2290)	20	1430 ± 140 (1340 - 2030)	1410 ± 50 (1330 - 1540)	1410 ± 40 (1360 - 1540)	1400 ± 50 (1310 - 1510)
Na in mg/L	21	1680 ± 70 (1610 - 1930)	20	1660 ± 50 (1610 - 1800)	1680 ± 60 (1570 - 1830)	1680 ± 50 (1590 - 1850)	1670 ± 60 (1550 - 1810)
NH_4^+ in mg N/L	21	2110 ± 60 (2020 - 2300)	20	2100 ± 70 (2020 - 2280)	2130 ± 80 (2000 - 2330)	2130 ± 69 (2020 - 2350)	2110 ± 80 (1960 - 2300)
NO_3^- in mg N/L	21	2080 ± 40 (2020 - 2160)	20	2120 ± 70 (1980 - 2330)	2110 ± 70 (1890 - 2230)	2120 ± 50 (2010 - 2200)	2130 ± 90 (1900 - 2330)
PO_4^{3-} in mg P/L	21	199 ± 10 (179 - 215)	20	181 ± 17 (127 - 202)	199 ± 11 (177 - 216)	198 ± 12 (175 - 217)	187 ± 16 (158 - 217)
SO_4^{2-} in mg/L	21	745 ± 22 (705 - 783)	20	755 ± 27 (715 - 811)	758 ± 31 (688 - 812)	731 ± 111 (258 - 807)	756 ± 37 (652 - 848)

Table S 10 Operation parameters and concentrations measured over time in the effluent of sampling points H2.1, H2.2, H2.3 and H2.4 (fine GAC). All values are rounded to three significant digits and given as average (AV) with standard deviation (SD), minimal and maximal values and the number of measured samples over time (n) used to calculate AV and SD.

	n	H2.1<br>EBCT = 24 min* Av. ± SD (min - max)	H2.2<br>EBCT = 68 min* Av. ± SD (min - max)	H2.3<br>EBCT = 91 min* Av. ± SD (min - max)	H2.4<br>EBCT = 113 min* Av. ± SD (min - max)
T in °C	8	20.3 ± 0.6 (19.5 - 21.3)	20.4 ± 0.7 (19.5 - 21.3)	20.4 ± 0.7 (19.5 - 21.3)	20.5 ± 0.7 (19.5 - 21.3)
pH	8	5.0 ± 0.4 (4.60-05.6)	6.3 ± 0.2 (6.1 - 6.6)	5.9 ± 0.3 (5.5 - 6.4)	6.0 ± 0.3 (5.6 - 6.4)
DOC in mg/L	20	70.8 ± 17.7 (39.1 - 128)	56.4 ± 24.2 (19.7 - 104)	46.3 ± 21.1 (16.6 - 88.4)	38.9 ± 16.8 (16.4 - 74.8)
UV_{265} in AU	20	0.23 ± 0.03 (0.13 - 0.29)	0.17 ± 0.08 (0.04 - 0.27)	0.14 ± 0.07 (0.03 - 0.23)	0.11 ± 0.05 (0.03 - 0.19)
Ca in mg/L	9	20.8 ± 30 (15.8 - 26.1)	20.2 ± 5.8 (14.4 - 29.3)	21.0 ± 4.3 (13.5 - 26.8)	20.8 ± 4.4 (13.8 - 28.9)
Cl in mg/L	8	20.8 ± 30 (15.8 - 26.1)	20.2 ± 5.8 (14.4 - 29.3)	21.0 ± 4.3 (13.5 - 26.8)	20.8 ± 4.4 (13.8 - 28.9)
K in mg/L	20	3060 ± 140 (2810 - 3310)	2990 ± 90 (2790 - 3190)	2950 ± 80 (2740 - 3070)	2960 ± 200 (2290 - 3340)
Na in mg/L	20	1390 ± 50 (1270 - 1480)	1390 ± 90 (1150 - 1540)	1430 ± 70 (1340 - 1610)	1420 ± 50 (1340 - 1540)
NH_4^+ in mg N/L	20	1660 ± 70 (1530 - 1780)	1650 ± 120 (1340 - 1840)	1710 ± 90 (1600 - 2020)	1690 ± 60 (1590 - 1810)
NO_3^- in mg N/L	20	2090 ± 90 (1940 - 2270)	2110 ± 140 (1750 - 2330)	2160 ± 110 (2040 - 2500)	2150 ± 90 (2010 - 2300)
PO_4^{3-} in mg P/L	20	2190 ± 100 (2010 - 2420)	2140 ± 60 (2020 - 2250)	2130 ± 50 (1980 - 2190)	2130 ± 130 (1670 - 2300)
SO_4^{2-} in mg/L	20	151 ± 16 (121 - 184)	202 ± 12 (177 - 224)	189 ± 10 (167 - 202)	190 ± 10 (160 - 220)
T in °C	20	773 ± 35 (727 - 879)	771 ± 25 (733 - 819)	759 ± 23 (705 - 788)	760 ± 50 (610 - 860)

* To simplify the discussion of the results, the EBCTs of the coarse-grained GAC were used in the text.

S 7 Removal of pharmaceuticals during GAC treatment

In this section further information on the removal of the selected pharmaceuticals is given.

S 7.1 Pharmaceutical degradation in influent tank

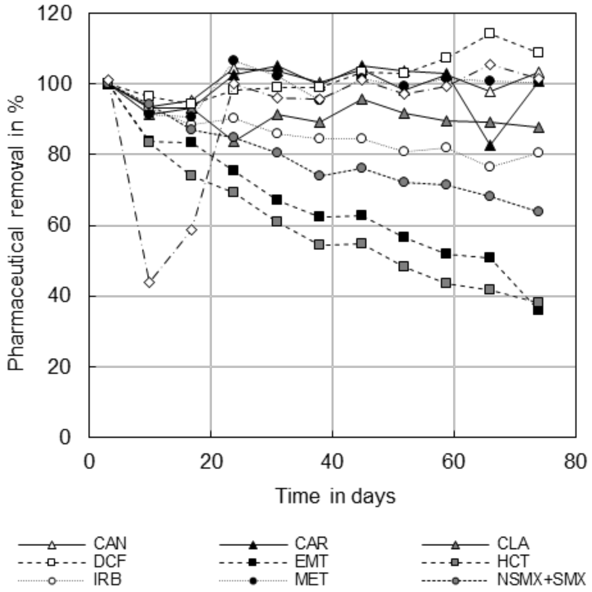

Figure S 9 Behavior of pharmaceuticals in the influent tank as a function of time

S 7.2 Pharmaceutical removal during treatment with GAC

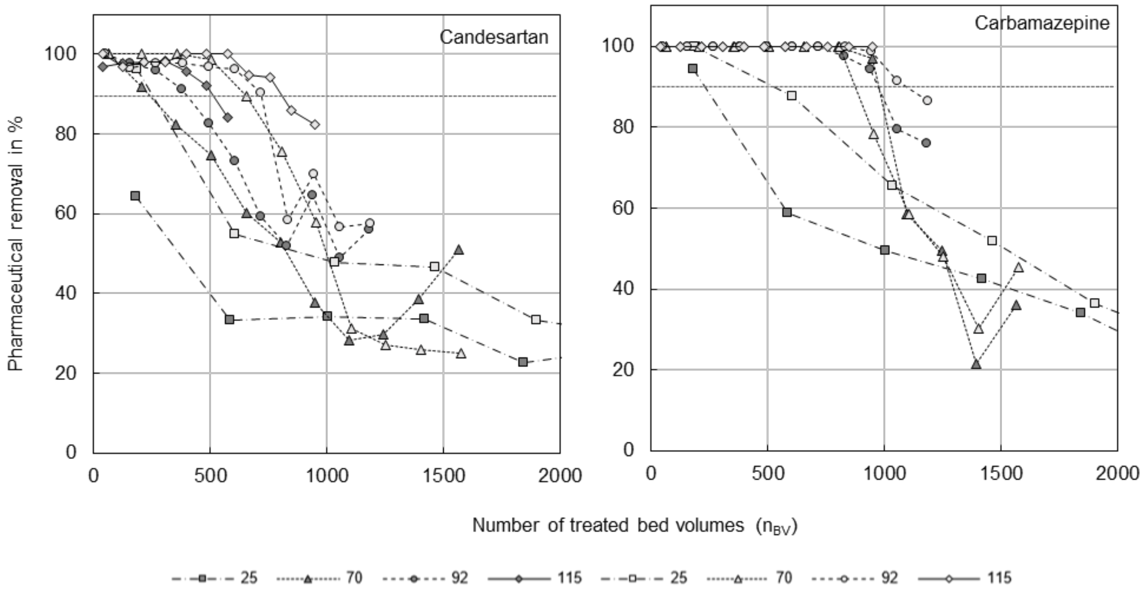

Figure S 10 Removal of candesartan and carbamazepine as a function of time for increasing empty bed contact times (in minutes) by adsorption on coarse (dark grey symbols) and fine (light grey symbols) GAC

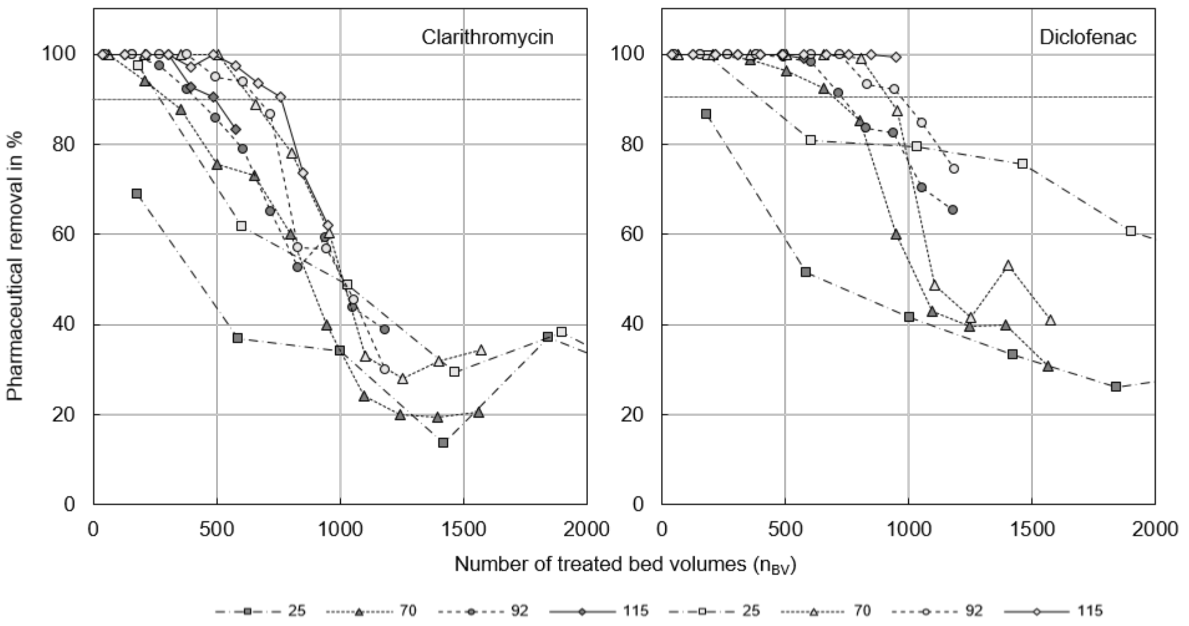

Figure S 11 Removal of clarithromycin and diclofenac as a function of time for increasing empty bed contact times (in minutes) by adsorption on coarse (dark grey symbols) and fine (light grey symbols) GAC

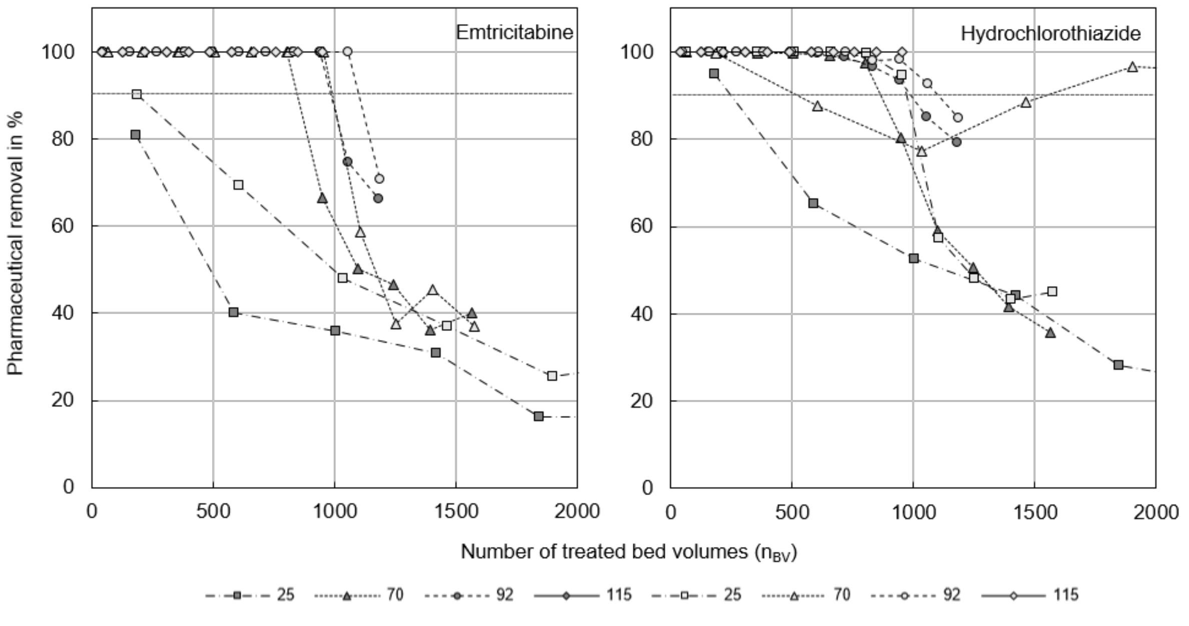

Figure S 12 Removal of emtricitabine and hydrochlorothiazide as a function of time for increasing empty bed contact times (in minutes) by adsorption on coarse (dark grey symbols) and fine (light grey symbols) GAC

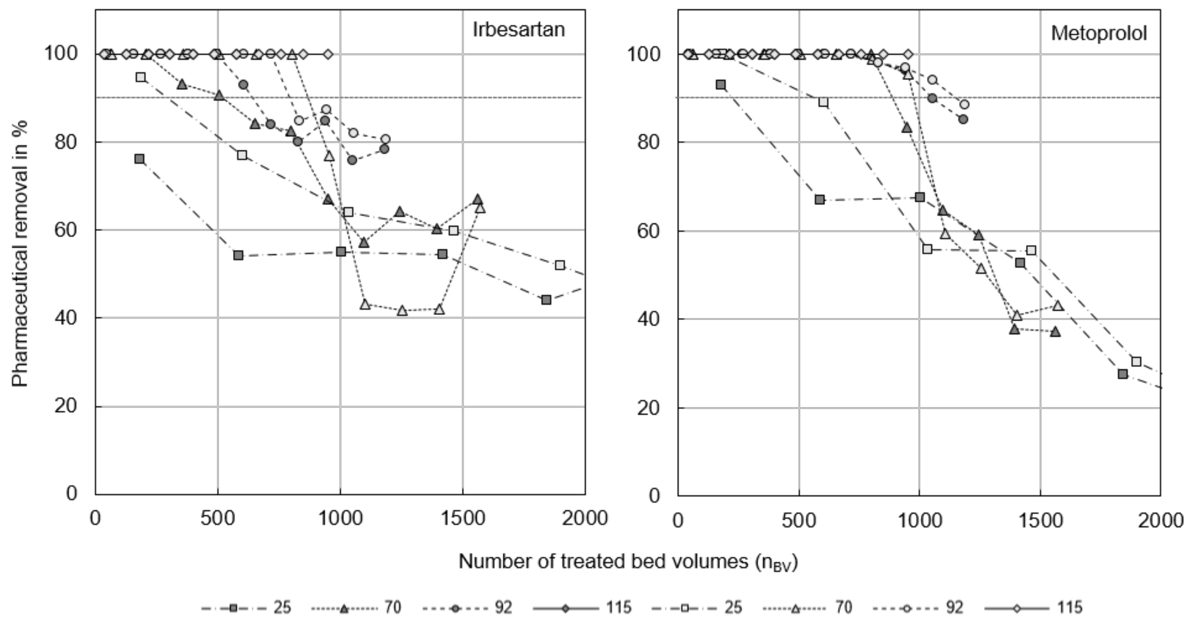

Figure S 13 Removal of irbesartan and metoprolol as a function of time for increasing empty bed contact times (in minutes) by adsorption on coarse (dark grey symbols) and fine (light grey symbols) GAC

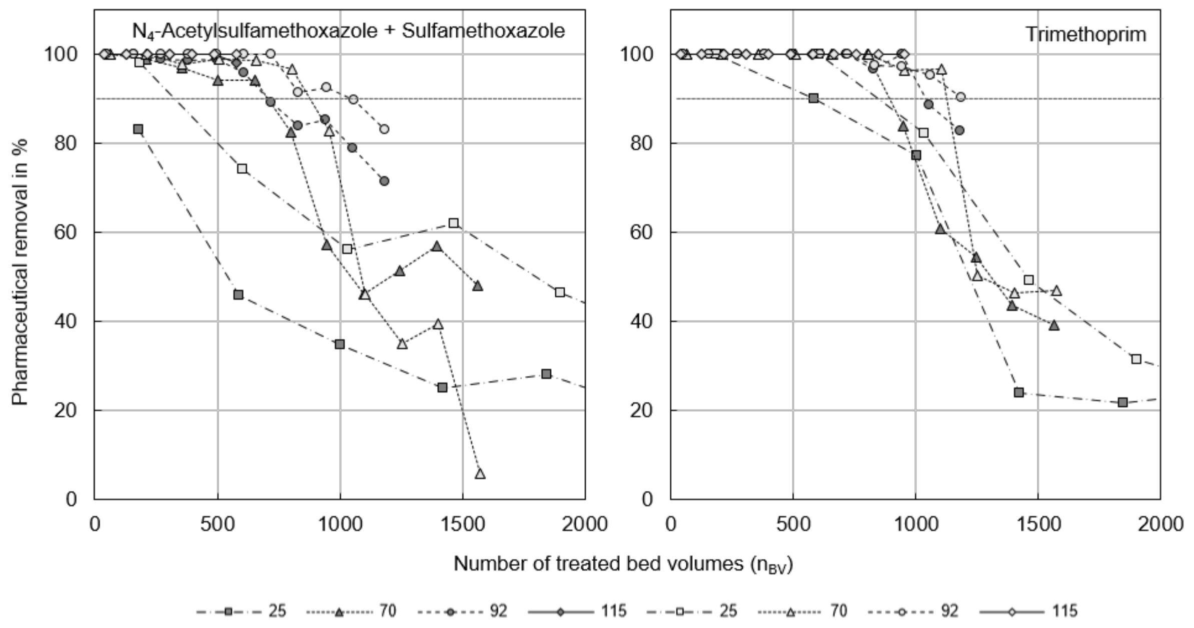

Figure S 14 Removal of N_4-acetylsulfamethoxazole+sulfamethoxazole and trimethoprim as a function of time for increasing empty bed contact times (in minutes) by adsorption on coarse (dark grey symbols) and fine (light grey symbols) GAC

S 7.3 Overall removal of investigated pharmaceuticals

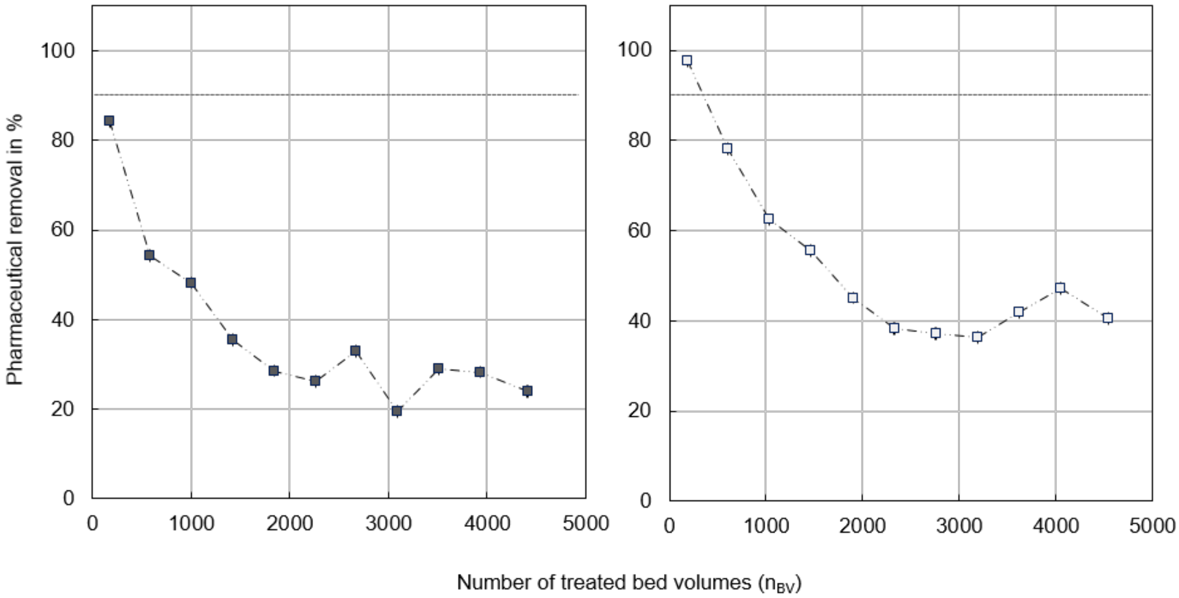

Figure S 15 Overall removal as the average of all pharmaceuticals with coarse (left) and fine (right) GAC as a function of the number of treated bed volumes (n_{BV}) for an empty bed contact times of 25 minutes

Table S 11 Comparison of the overall pharmaceutical removal calculated as mean and as median for empty bed contact times (EBCT) of 25, 70, 92 and 115 minutes for the treatment with coarse GAC and presented with the corresponding number of treated bed volumes (n_{BV})

25			70			92			115		
	Mean	Median		Mean	Median		Mean	Median		Mean	Median
n_{BV}	%	%	n_{BV}	%	%	n_{BV}	%	%	n_{BV}	%	%
182	84	84.8	64	100	100	49	100	100	39	100	100
587	54	52.8	209	98	100	158	100	100	127	100	100
1000	48	45.5	356	96	99.4	269	99	100	217	100	100
1420	35	33.4	504	93	97.9	381	98	100	307	100	100
1850	29	27.8	655	90	96.6	495	96	100	398	99	100
2260	26	24.2	801	86	91.3	606	93	98.2	487	98	100
2680	33	30.2	949	67	66.8	718	88	91.5	577	96	100
3090	19	18.7	1100	49	53.8	830	83	83.8	667	n.a.*	n.a.
3510	29	24.7	1250	47	50.1	942	85	85.2	757	n.a.	n.a.
3930	28	25.8	1390	40	39.3	1050	72	75.8	848	n.a.	n.a.
4410	24	17.1	1560	41	38.3	1180	69	71.4	951	n.a.	n.a.

*n.a. stands for not analyzed

Table S 12 Comparison of the overall pharmaceutical removal calculated as mean and as median for empty bed contact times (EBCT) of 25, 70, 92 and 115 minutes for the treatment with fine GAC and presented with the corresponding number of treated bed volumes (n_{BV})

25			70			92			115		
	Mean	Median		Mean	Median		Mean	Median		Mean	Median
n_{BV}	%	%	n_{BV}	%	%	n_{BV}	%	%	n_{BV}	%	%
187	98	98.9	65	100	100	49	100	100	39	100	100
605	78	78.9	209	100	100	158	100	100	127	100	100
1040	63	60.0	358	100	100	270	100	100	217	100	100
1470	56	53.7	508	100	100	383	100	100	307	100	100
1900	45	37.4	659	98	100	496	99	100	398	100	100
2330	38	29.1	806	95	99.5	608	99	100	488	100	100
2760	37	29.0	955	83	85.1	720	98	100	578	100	100
3190	36	28.2	1100	53	53.1	832	88	95.4	668	99	100
3620	42	34.1	1250	41	41.7	945	89	94.7	758	98	100
4050	47	42.2	1400	40	41.4	1060	83	90.6	849	96	100
4540	41	36.2	1570	39	42.1	1190	75	81.8	952	94	100

S 7.4 Calculation of specific surface

In this section the calculation of the outer specific surface of the coarse and the fine GAC is explained. This value was used to compare the elimination efficiencies of the two GAC grain sizes.

The average particle diameters for coarse and fine GAC are assumed to be 1.9 mm and 0.8 mm, respectively. Assuming, that the granules are spheres, the volume (*V*) and surfaces (*A*) are:

$$V = \frac{4}{3}\pi \cdot r^3 \quad (10)$$

and

$$A = 4\pi \cdot r^2 \quad (11)$$

We obtained:

$V_{coarse\ particle} = 3.59\ mm^3$ and $A_{coarse\ particle} = 11.3\ mm^2$ as well as

$V_{fine\ particle} = 0.268\ mm^3$ and $A_{fine\ particle} = 2.01\ mm^2$

$V_{reactor,coarse} = 146{,}000\ mm^3$ and $V_{reactor,fine} = 145{,}000\ mm^3$

To calculate the total volume of both GACs (V_{coarse} and $V_{fine,}$, respectively), the fraction of the volume taken by the GAC particles was multiplied with the reactor volume ($V_{reactor}$). The fraction of the volume taken by the GAC particles was calculated by subtracting the bed porosity (ε) from 1. The bed porosity of each GAC bed was determined before starting the experiment (see Table S 13). For this, the columns

were filled with water up to the upper level of the GAC bed and then the water was drained through the bottom valve and the volume of the pore water (V_{pore}) was noted. Dividing the obtained pore volume by the volume of the GAC bed ($V_{GAC,\ bed}$) gives the porosity.

$$V_{coarse} = (1-\varepsilon)\cdot V_{reactor} = (1-0.43)\cdot 1{,}460{,}000\ mm^3 = 832{,}200\ mm^3 \quad (12)$$

$$V_{fine} = (1-\varepsilon)\cdot V_{reactor} = (1-0.39)\cdot 1{,}450{,}000\ mm^3 = 884{,}500\ mm^3 \quad (13)$$

The number of coarse particles, n_{coarse}, is obtained by

$$n_{coarse} = \frac{V_{coarse}}{V_{coarse\ particle}} = 231{,}811 \quad (14)$$

And the number of fine particles, n_{fine}, is obtained by

$$n_{fine} = \frac{V_{fine}}{V_{fine\ particle}} = 3{,}300{,}373 \quad (15)$$

The total surface of the coarse and the fine GAC are

$$A_{coarse} = n_{coarse}\cdot A_{coarse\ particle} = 231{,}811\cdot 11.3\ mm^2 = 2{,}619{,}460\ mm^2 \quad (16)$$

$$A_{fine} = n_{fine}\cdot A_{fine\ particle} = 3{,}300{,}373\cdot 2.01\ mm^2 = 6{,}633{,}750\ mm^2 \quad (17)$$

The ratio of the total surface of coarse and fine GAC becomes

$$\frac{A_{fine}}{A_{coarse}} = 2.53 \quad (18)$$

S 8 Comparison with advanced wastewater treatment

In this section additional information on the comparison with the GAC treatment of wastewater treatment plan (WWTP) effluent is given. Numbers were rounded to three significant digits.

Table S 14 General information on the influent characteristics and the GAC treatment of the studies

Study		Bourgin et al. (2018) Wastewater 1	Wunderlin et al. (2017) Wastewater 2	This study Nitrified urine
Medium		biologically treated municipal wastewater	biologically treated municipal wastewater	source-separated, nitrified urine
pH	-	6.8-7.9 (7.6)		6.9
T	°C	13-23		19
DOC	mg/L	5.31	5.50	103
Total N	mgN/L	6.78		4060
NH_4^+	mgN/L	0.08		2020
NO_3^-	mgN/L	6.7		2040
Reactor height	cm			20.3
Inner diameter	cm			5.36
Empty bed volume	L	77	32600	0.458
Flow rate	L/h	300		0.3
Filter velocity	m/h		4.6	0.14
EBCT	min	14	21	91
GAC type		Cyclecarb 401, Chemviron	Aquasorb 5010, Jacobi	Norit® GCN830, Norit
GAC grain size	mm	0.43 - 2.36	1.2-2.3 mm	0.6 - 1.0 mm
GAC mass	g	34,700	12,900,000	267
Specific GAC mass	kg/m^3	450	395	583

Table S 15 Influent concentrations (c_{inf}) of dissolved organic carbon (DOC) and the investigated pharmaceuticals

c_{inf} in µg/L	Wastewater 1	Wastewater 2	Nitrified urine
CAN	0.34	0.90	11.1
CAR	0.19	0.42	5.37
CLA	0.29	0.26	47.4
DCF	1.36	2.42	82.4
EMT			1.70
HCT	0.99	1.09	51.3
IRB	0.50	0.85	4.05
MET	0.27	0.38	26.8
NSMX			3.21
SMX	0.10	0.40	5.53
TMP			4.13
DOC in mg/L	5.31	5.50	103

Table S 16 Number of treated bed volumes (n_{BV}) calculated for a removal goal of ≥ 90%. Numbers are rounded to three significant digits.

n_{BV} in m^3/m^3	Wastewater 1	Wastewater 2	Nitrified urine
CAN	2530	734	832
CAR	7390	11100	1190
CLA	7390	3510	720
DCF	7390	1820	1060
EMT			1190
HCT	7390	19500	1190
IRB	4370	2650	832
MET	7390	19500	1190
NSMX			1190
SMX	2530	2140	1060
TMP			1190
Average	5800	7620	1040

Table S 17 Total volume treated ($V_{treated}$) calculated for a removal goal of ≥ 90%. Numbers are rounded to three significant digits.

V_{total} in m^3	Wastewater 1	Wastewater 2	Nitrified urine
CAN	195	23,900	0.381
CAR	569	361,000	0.543
CLA	569	115,000	0.330
DCF	569	59,300	0.485
EMT			0.543
HCT	569	637,000	0.543
IRB	337	86,500	0.381
MET	569	637,000	0.543
NSMX			0.543
SMX	195	69,700	0.485
TMP			0.543
Average	447	249,000	0.484

Table S 18 Total amount of adsorbed compound ($m_{pharma,\ adsorbed}$) calculated for a removal goal of ≥ 90%. Numbers are rounded to three significant digits.

$m_{pharma,adsorbed}$ in mg	Wastewater 1	Wastewater 2	Nitrified urine
CAN	66	21,700	4.2
CAR	107	153,000	2.9
CLA	165	30,200	15.6
DCF	771	144,000	39.9
EMT			0.9
HCT	563	693,000	27.9
IRB	167	73,100	1.5
MET	155	244,000	14.6
NSMX			1.7
SMX	19	28,000	2.7
TMP			2.2
Total	2013	1,387,000	114

Table S 19 Calculated carbon usage rates (CUR) calculated for a removal goal of ≥ 90%

CUR in mg GAC/L	Wastewater 1	Wastewater 2	Nitrified urine
CAN	178	538	701
CAR	61	36	492
CLA	61	112	810
DCF	61	217	551
EMT			492
HCT	61	20	492
IRB	103	149	701
MET	61	20	492
NSMX			492
SMX	178	185	551
TMP			492
Average	95	160	569

Table S 20 Required amount of carbon related to the influent DOC calculated for a removal goal of ≥ 90%

m_{GAC}/m_{DOC} in mg GAC/mg $DOC_{influent}$	Wastewater 1	Wastewater 2	Nitrified urine
CAN	33	98	6.8
CAR	11	6	4.8
CLA	11	20	7.9
DCF	11	40	5.3
EMT			4.8
HCT	11	4	4.8
IRB	19	27	6.8
MET	11	3.7	4.8
NSMX			4.8
SMX	33	34	5.3
TMP			4.8
Average	18	29	5.5

Table S 21 Daily required amount of carbon per person calculated for a removal goal of ≥ 90%

m_{GAC}/person/day	Wastewater 1	Wastewater 2	Nitrified urine
CAN	49	147	0.88
CAN	49	147	0.88
CAR	17	10	0.61
CLA	17	31	1.01
DCF	17	60	0.69
EMT			0.61
HCT	17	6	0.61
IRB	28	41	0.88
MET	17	6	0.61
NSMX			0.61
SMX	49	51	0.69
TMP			0.61
Average	26	44	0.71

S 8.1 Calculation of personal CUR

CURs calculated for a daily wastewater production of 350 L:

$$\varnothing CUR_{WW1} = 95\frac{mg\ GAC}{L}\cdot 350\frac{L}{person\cdot day} = 33\ \frac{g\ GAC}{person\cdot day} \tag{19}$$

$$\varnothing CUR_{WW2} = 160\frac{mg\ GAC}{L}\cdot 350\frac{L}{person\cdot day} = 56\ \frac{g\ GAC}{person\cdot day} \tag{20}$$

$$\varnothing CUR_{nitrified\ urine} = 569\frac{mg\ GAC}{L}\cdot 1.25\frac{L}{person\cdot day} = 0.7\ \frac{g\ GAC}{person\cdot day} \tag{21}$$

The GAC demand for pharmaceutical removal in this example is 60 times or nearly two orders of magnitude smaller for urine treatment than for the treatment of WWTP effluent.

CURs calculated for a daily wastewater production of 200 L:

$$\emptyset CUR_{WW1} = 95\frac{mg\ GAC}{L} \cdot 200\frac{L}{person{\cdot}day} = 19\ \frac{g\ GAC}{person{\cdot}day} \tag{22}$$

$$\emptyset CUR_{WW2} = 160\frac{mg\ GAC}{L} \cdot 200\frac{L}{person{\cdot}day} = 32\ \frac{g\ GAC}{person{\cdot}day} \tag{23}$$

$$\emptyset CUR_{nitrified\ urine} = 569\frac{mg\ GAC}{L} \cdot 1.25\frac{L}{person{\cdot}day} = 0.7\ \frac{g\ GAC}{person{\cdot}day} \tag{24}$$

The GAC demand for pharmaceutical removal in this example is 36 times or nearly two orders of magnitude smaller for urine treatment than for the treatment of WWTP effluent.

S 8.2 Influence of urine nutrients by GAC treatment

In this section additional information on the effect of the GAC treatment on the urine nutrients is given.

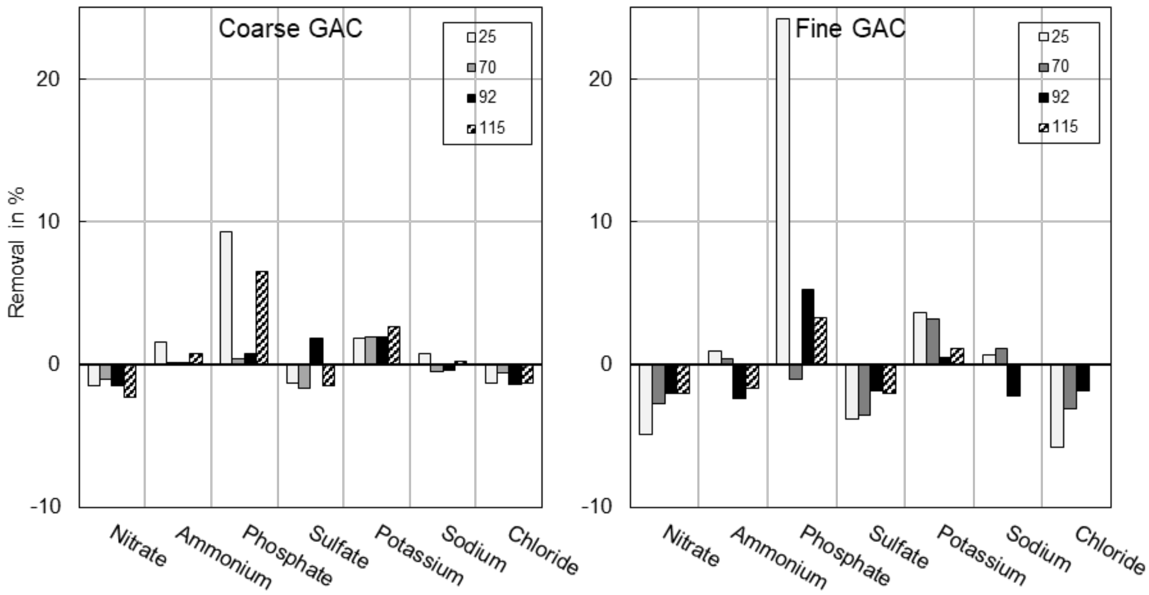

Figure S 16 Removal of urine nutrients by treatment with coarse (left) and fine (right) GAC at different EBCTs in minutes. The presented values are averages of 21 grab samples taken every third day during the entire duration of the experiment.

S 9 Local removal of phosphate

For fine-grained GAC we observed a local anomaly of the phosphate concentration at the sampling port for EBCT = 25 min. At this point, phosphate was removed on average by almost 25%. On day 3, 10% were eliminated and 38% on day 32 (Figure S 17, left). However, the phosphate removal was a local phenomenon. At the following sampling points, phosphate concentrations were higher. At the sampling port for EBCT = 115 min, the phosphate concentration was 3.3% lower than the phosphate concentration in the influent. The phosphate concentrations correlated with the pH values. The effluent pH at the sampling port for EBCT = 25 min decreased by 18 to 28% compared to the influent pH (Figure S 17, right). The minimum pH of 4.6 was measured on days 59 and 70. The pH was always higher at later sampling points and reached similar values as in the influent. In addition to the drop of the pH and the phosphate concentration, we observed white stains in the GAC bed around the nozzle of the sampling port for EBCT = 25 min (Figure S 18) and a significant reduction of the flow velocity during sampling. At the end of the experiment, yellow-whitish depositions on the in- and outside of this nozzle were found. The observations we made at the sampling port for EBCT = 25 min, were most probably due to nitrification by acid-tolerant ammonium oxidizing bacteria, leading to brass corrosion and local precipitation of metal phosphates. Acid-tolerant ammonium oxidizing bacteria were previously observed to grow in urine nitrification reactors, when the influent was switched off but aeration continued (Fumasoli et al., 2017). We assume that by adding nitrified urine to the top of the GAC columns sufficient oxygen was provided for the growth of acid-tolerant ammonium oxidizing bacteria. The pH decrease triggered the corrosion of the brass nozzles. The high concentrations of chloride (2900 mg/L) and sulfate (745 mg/L) as well as the high concentration of ammonia (2130 mg/L), in combination with the little carbonate hardness of nitrified urine, were reported to be a corrosive environment for brasses (Namboodhiri et al., 1982, and Dinnappa and Mayanna, 1987, respectively).

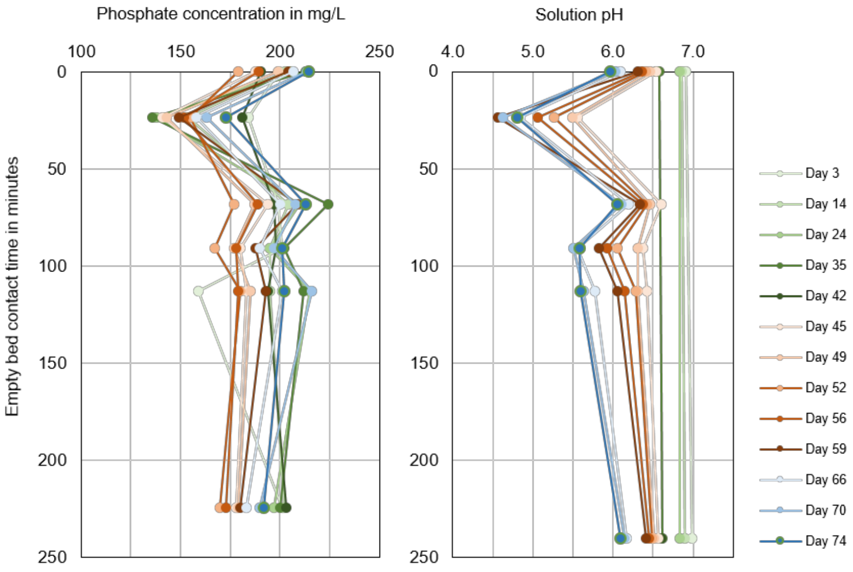

Figure S 17 Measured effluent phosphate concentration and solution pH as a function of time and in dependence of the empty bed contact time in minutes for the treatment with fine GAC

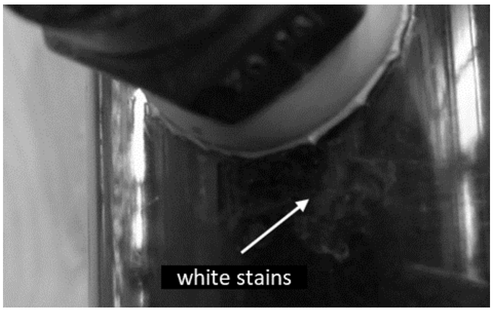

Figure S 18 Precipitation we observed at the end of the experiment around the first sampling nozzle (EBCT = 25 min) in the column filled with fine GAC

S 9.1 Batch experiments to investigate the fate of dissolved phosphate

We conducted two lab-scale batch experiments to investigate the role of the low pH and the corrosion of the sampling port on the dissolved phosphate concentration. The sampling port of sampling point H2.1 was used for the experiments. In the second experiment the number of samples and the reaction times were increased – the experimental procedure was the same. We will show the results of the second batch experiment and the analysis of the solid samples.

S 9.2 Experimental procedure

The batch experiments were conducted as follows: The sampling port was immersed in 322.85 mg nitrified urine, which was collected from the nitrification reactor in the basement of Forum Chriesbach (Eawag) and placed on a magnetic stirrer. The solution pH was measured continuously. After 30 minutes, the pH was set to a value of 5.0 by adding 7.3 mL HNO_3 (0.1 M). The solution was kept for one hour and the first sample (sample 1, pH 5, V = 50 mg) for solids analysis was taken after 60 minutes. After sampling, the pH was increased again to 7.0 by the addition of 0.7 mL NaOH (4%) and was left for reaction. After five hours, the second sample (sample 2, pH 7, V = 153 mg) for solids analysis was taken and the experiment was stopped.

S 9.2.1 Solid analysis

The suspended solids concentration (TSS) of the samples were 44 and 42 mg/L for samples 1 and 2, respectively. The samples were filtered with a cellulose acetate filter (OE67, pore size: 0.45µm, Whatman, Maidstone, United Kingdom), then dissolved with 65% HNO_3, and finally analyzed with inductively coupled plasma optical emission spectrometry (ICP-OES) (Arcos, Spectro, 47533 Kleve, Germany). With this, contents of boron (B), calcium (Ca), copper (CU), iron (Fe), potassium (K), magnesium (Mg), molybdenum (Mo), sodium (Na), nickel (Ni), lead (Pb), strontium (Sr), thallium (Ti) and zinc (Zn) were determined.

S 9.2.2 Results

The dissolved phosphate concentration directly decreased after the start of the experiment from initially 146 mg/L to about 115 mg/L, and later on stayed almost constant until the end of the experiment (Figure

S 19). Meanwhile, turbidity of the solution changed from clear to milky, indicating ongoing precipitation processes. The adaption of the solution pH from initially 6.45 to 5.0 and back to 7.0 did not seem to affect the dissolved phosphate concentration. Precipitates were observed in both samples during sampling. The metal composition and their concentrations were different for samples 1 and 2. Solids taken at pH 5 showed higher concentrations of Cu and Fe, while Zn, Ni and Pb concentrations were higher in the solids taken at pH 7 (Figure S 20).

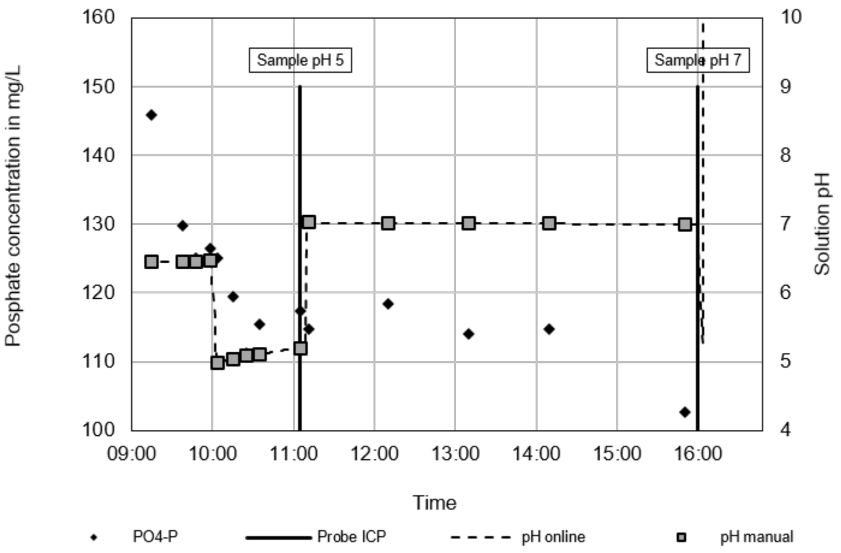

Figure S 19 Dissolved phosphate concentration and pH as a function of time during batch experiment 2

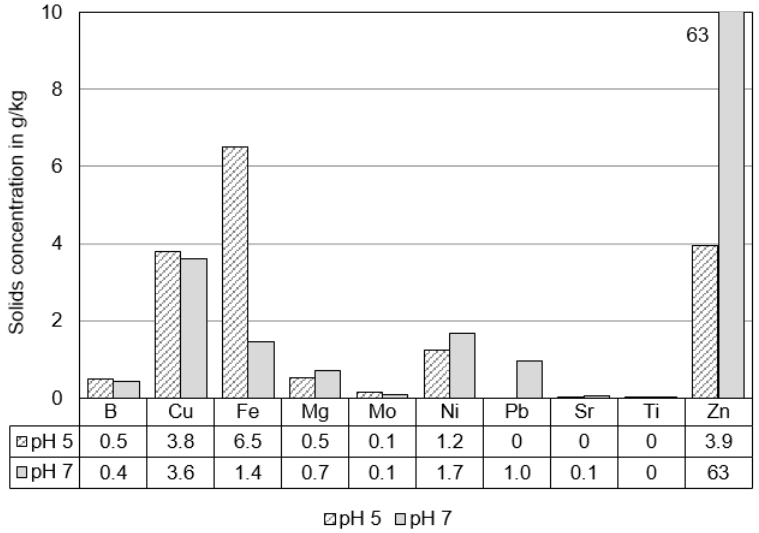

	B	Cu	Fe	Mg	Mo	Ni	Pb	Sr	Ti	Zn
pH 5	0.5	3.8	6.5	0.5	0.1	1.2	0	0	0	3.9
pH 7	0.4	3.6	1.4	0.7	0.1	1.7	1.0	0.1	0	63

pH 5 pH 7

Figure S 20 Results of ICP-OES analysis for solid samples taken at pH 5 and pH 7 during batch experiment

S 10 UV_{265} removal and DOC removal as surrogate parameter for pharmaceutical removal

In this section additional information on the UV_{265} removal and DOC removal are given.

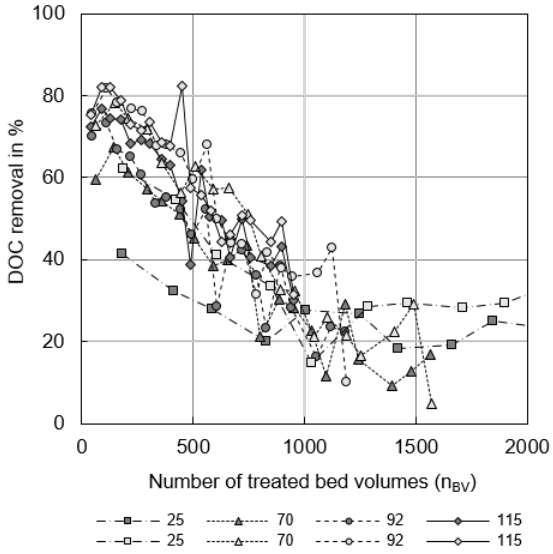

Figure S 21 DOC removal as a function of the number of treated bed volumes at EBCTs of 25, 70, 92 and 115 minutes for the adsorption on coarse (dark grey symbols) and fine (light grey symbols) GAC

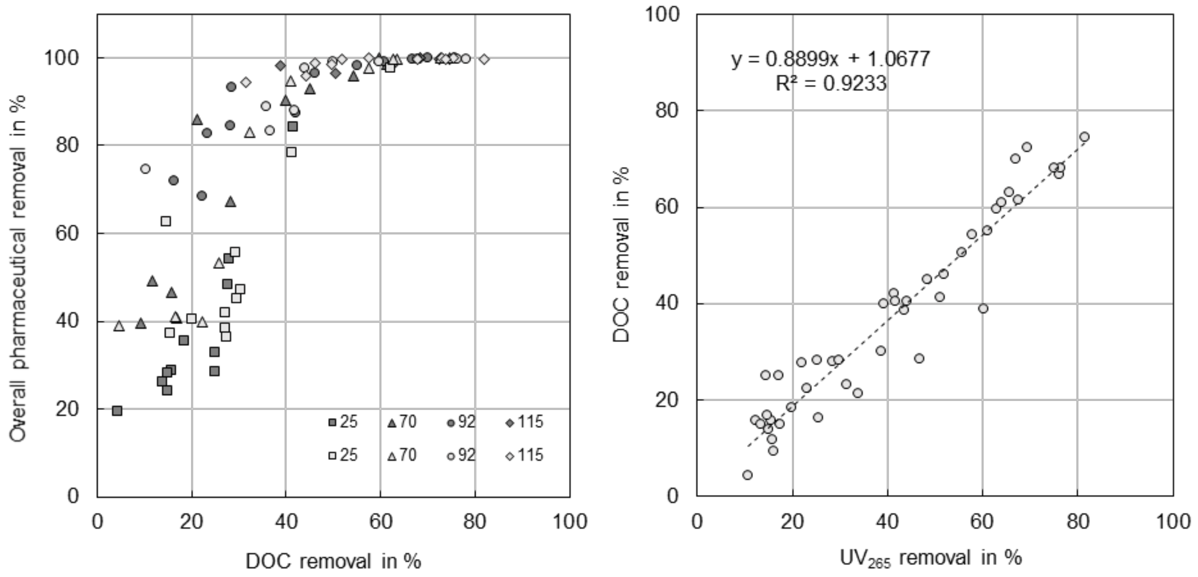

Figure S 22 Relationship between DOC removal and UV_{265} removal in nitrified urine after treatment with GAC. Data points include all EBCTs for coarse (dark grey symbols) and fine (light grey symbols) GAC.

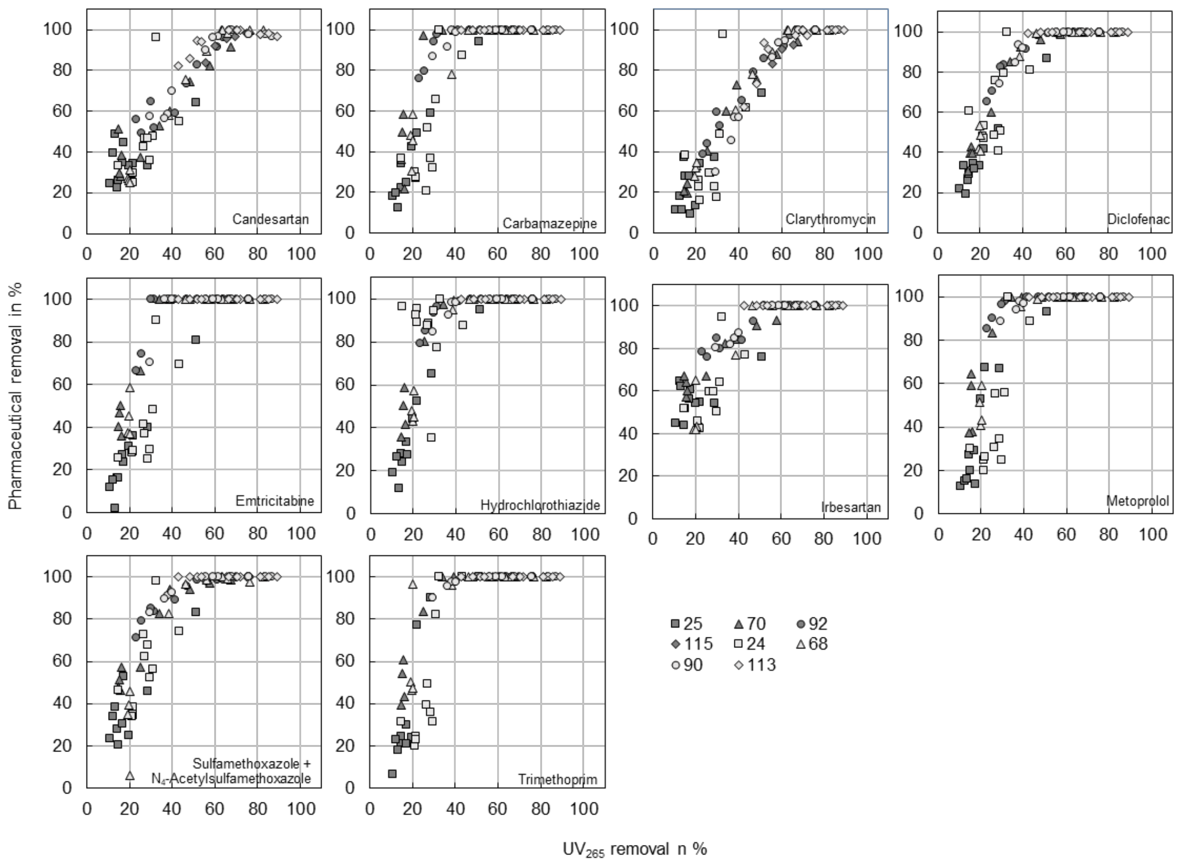

Figure S 23 **Pharmaceutical removal as a function of UV_{265} removal at empty bed contact times of 25, 70, 92 and 115 minutes for the adsorption on coarse GAC (dark grey symbols) and at 24, 68, 90 and 113 minutes for the adsorption on fine GAC (light grey symbols).**

References

Altmann, J., Massa, L., Sperlich, A., Gnirss, R. and Jekel, M. (2016) UV_{254} absorbance as real-time monitoring and control parameter for micropollutant removal in advanced wastewater treatment with powdered activated carbon. Water Research 94, 240-245.

Bourgin, M., Beck, B., Boehler, M., Borowska, E., Fleiner, J., Salhi, E., Teichler, R., von Gunten, U., Siegrist, H. and McArdell, C.S. (2018) Evaluation of a full-scale wastewater treatment plant upgraded with ozonation and biological post-treatments: Abatement of micropollutants, formation of transformation products and oxidation by-products. Water Research 129, 486-498.

Dinnappa, R.K. and Mayanna, S.M. (1987) The dezincification of brass and its inhibition in acidic chloride and sulphate solutions. Corrosion Science 27(4), 349-361.

Fumasoli, A., Burgmann, H., Weissbrodt, D.G., Wells, G.F., Beck, K., Mohn, J., Morgenroth, E. and Udert, K.M. (2017) Growth of Nitrosococcus-related ammonia oxidizing bacteria coincides with extremely low pH values in wastewater with high ammonia content. Environmental Science & Technology 51(12), 6857-6866.

Götz, C., Otto, J. and Singer, H. (2014) Substanzen zur Überprüfung des Reinigungseffekts weitergehender Abwasserbehandlungsverfahren. Fachbericht im Auftrag des Bundesamts für Umwelt BAFU. Eawag, Dübendorf, Switzerland. Substances to control the cleaning effectivity of advanced wastewater treatment processes. Technical report on behalf of the Swiss Federal Office of Environment (FOEN).

Kårelid, V., Larsson, G. and Björlenius, B. (2017) Pilot-scale removal of pharmaceuticals in municipal wastewater: Comparison of granular and powdered activated carbon treatment at three wastewater treatment plants. Journal of Environmental Management 193(Supplement C), 491-502.

Crittenden, J.C., Trussell, R.R., Hand, D.W., Howe, K.J., Tchobanoglous, G. (2012) MWH's Water Treatment: Principles and Design, Third Edition, Wiley and Sons, pp. 1117-1262.

Namboodhiri, T.K.G., Chaudhary, R.S., Prakash, B. and Agrawal, M.K. (1982) The dezincification of brasses in concentrated ammonia. Corrosion Science 22(11), 1037-1047.

Wunderlin, P., Joss, A. and Fleiner, J. (2017) Elimination von Spurenstoffen durch granulierte Aktivkohle (GAK) Filtration: Grosstechnische Untersuchungen auf der ARA Bülach-Furt. Zwischenbericht. Eawag, Dübendorf. Elimination of micropollutants by granular acticated carbon (GAC) filtration: Pilot-scale study on WWTP Bülach-Furt. Interim report.
